# Cotrimoxazole Prophylaxis Increases Resistance Gene Prevalence and α-Diversity but Decreases β-Diversity in the Gut Microbiome of Human Immunodeficiency Virus–Exposed, Uninfected Infants

**DOI:** 10.1093/cid/ciz1186

**Published:** 2019-12-13

**Authors:** Alaric W D’Souza, Eshia Moodley-Govender, Bertram Berla, Tejas Kelkar, Bin Wang, Xiaoqing Sun, Brodie Daniels, Anna Coutsoudis, Indi Trehan, Gautam Dantas

**Affiliations:** 1 Edison Family Center for Genome Sciences and Systems Biology, Washington University in St Louis School of Medicine, St Louis, Missouri, USA; 2 Department of Paediatrics and Child Health, University of KwaZulu-Natal, Durban, South Africa; 3 Department of Pathology and Immunology, Washington University in St Louis School of Medicine, St Louis, Missouri, USA; 4 HIV Preventin Research Unit, South African Medical Research Council, Durban, South Africa; 5 Department of Pediatrics, Washington University in St Louis School of Medicine, St Louis, Missouri, USA; 6 Department of Molecular Microbiology, Washington University in St Louis School of Medicine, St Louis, Missouri, USA; 7 Department of Biomedical Engineering, Washington University in St Louis, St Louis, Missouri, USA

**Keywords:** cotrimoxazole prophylaxis, antibiotic resistance, HIV-exposed, uninfected infant, microbiome

## Abstract

**Background:**

Prophylactic cotrimoxazole treatment is recommended in human immunodeficiency virus (HIV)–exposed, uninfected (HEU) infants, but the effects of this treatment on developing HEU infant gut microbiotas and resistomes are largely undefined.

**Methods:**

We analyzed whole-metagenome sequencing data from 163 longitudinally collected stool samples from 63 HEU infants randomized to receive (n = 34; CTX-T) or to not receive (n = 29; CTX-N) prophylactic cotrimoxazole treatment. We generated taxonomic, functional pathway, and resistance gene profiles for each sample and compared microbiome signatures between the CTX-T and CTX-N infants.

**Results:**

Metagenomic analysis did not reveal significant differences in taxonomic or functional pathway α-diversity between CTX-T and CTX-N infants. In contrast, resistance gene prevalence (*P* = .00719) and α-diversity (*P* = .0045) increased in CTX-T infants. These differences increased over time for both resistance gene prevalence measured by log-normalized abundance (4-month mean, 0.71 [95% confidence interval {CI}, .2–1.2] and 6-month mean, 0.85 [95% CI, .1–1.7]) and α-diversity (*P* = .0045). Unlike α-diversity, interindividual gut microbiome taxonomic (mean, −0.11 [95% CI, −.15 to −.077]), functional taxonomic (mean, −0.050 [95% CI, −.084 to −.017]), and resistance gene (mean, −0.13 [95% CI, −.17 to −.099]) β-diversity decreased in CTX-T infants compared with CTX-N infants. These results are consistent with persistent antibiotic selection pressure.

**Conclusions:**

Cotrimoxazole prophylaxis in HEU infants decreased gut microbiome β-diversity and increased antibiotic resistance gene α-diversity and prevalence. Antibiotic resistance is a growing threat, especially in low- and middle-income countries where the higher perinatal HIV exposure rates result in cotrimoxazole prophylaxis. Understanding effects from current HEU infant antibiotic prophylaxis guidelines will inform guideline revisions and efforts to reduce increasing antibiotic resistance.

## Teaser

Cotrimoxazole treatment in HIV-exposed, uninfected (HEU) infants increases resistance gene prevalence and α-diversity, and decreases microbial taxonomic, functional pathway, and resistance gene β-diversity. Future HEU infant prophylaxis recommendations should consider these findings given rising global antibiotic resistance.

**(See the Editorial Commentary by Bourke and Evans on pages 2869–71.)**

Infants perinatally exposed to human immunodeficiency virus (HIV) have higher infection risk after birth than HIV-unexposed infants [1–8]. This higher infection risk exists regardless of the infant’s HIV status [4, 8]. The World Health Organization (WHO) recommends prophylactic cotrimoxazole therapy for all HIV-positive adults and infants in areas with high prevalence of severe bacterial infections and malaria, and for HIV-exposed, uninfected (HEU) infants to prevent *Pneumocystis jirovecii* infection [9]. Cotrimoxazole is a broad-spectrum antibiotic coformulation of trimethoprim and sulfamethoxazole that inhibits bacterial tetrahydrofolic acid synthesis [10]. In HIV-infected infants, cotrimoxazole therapy reduces early-life infection due to pneumonia, diarrhea, and malaria [11] and may reduce systemic inflammation [12]. In malaria-endemic areas, cotrimoxazole therapy improves HEU infant survival, but in nonmalaria areas with routine HIV testing, cotrimoxazole therapy’s benefits may be outweighed by risk of gut dysbiosis and increased antimicrobial resistance [13–15].

WHO guidelines recommend that HEU infants receive prophylactic cotrimoxazole from 4–6 weeks after birth until breastfeeding ceases [9]. The guidelines say this is a “strong recommendation” with “very-low-quality evidence.” Since breast feeding is recommended until children are at least 2 years old [16], HEU infants receive cotrimoxazole prophylaxis through the critical period when the gut microbiota transitions from its nascent state to its mature composition [17–22]. Gut microbiota perturbations during infancy are linked to negative health consequences [23–25]. The WHO guidelines [9] note that cotrimoxazole prophylaxis in HEU infants “might cause gut perturbations and affect the gut microbiome,” but the details of these gut microbiome changes remain undefined.

**Figure 1.**
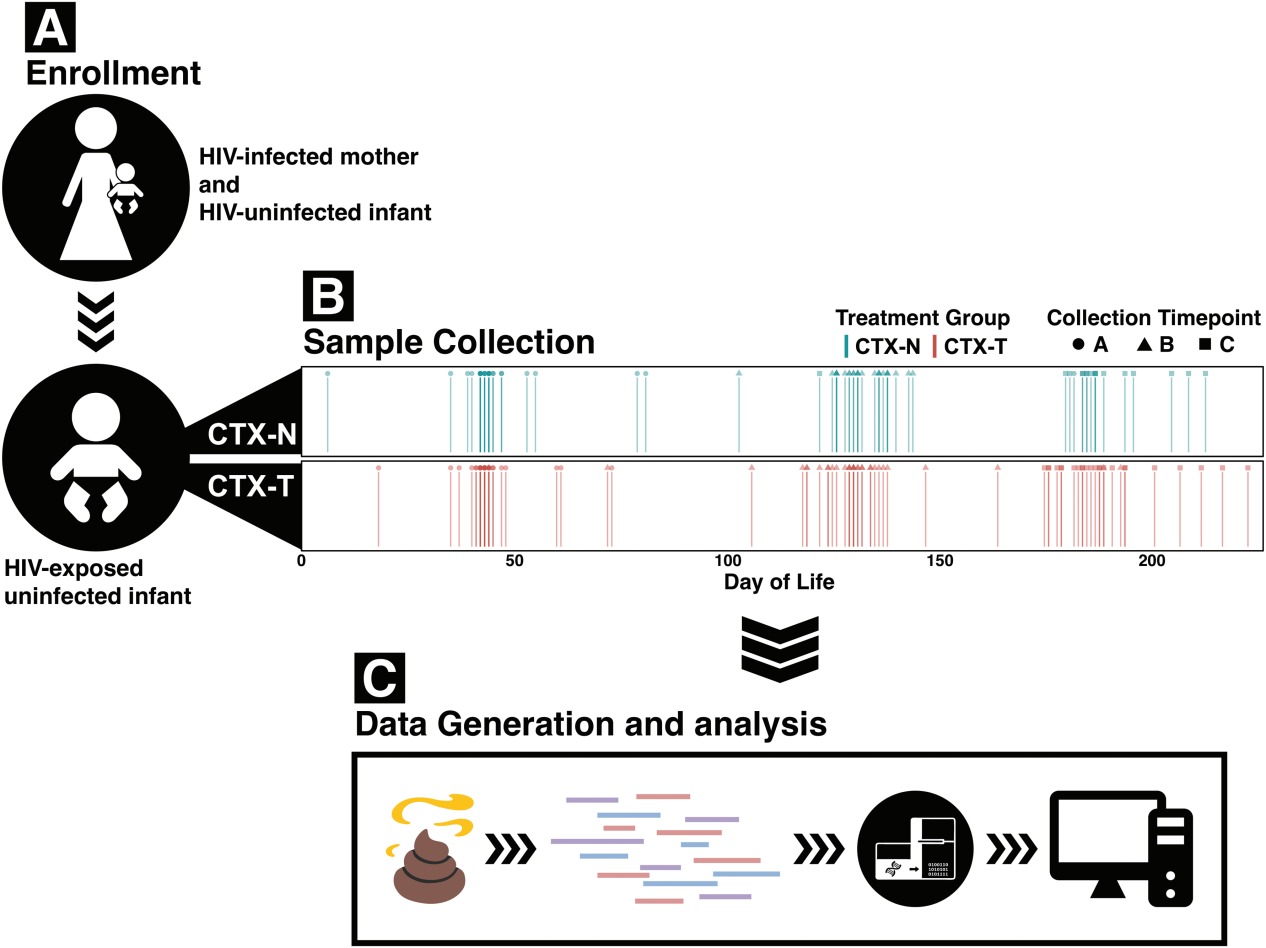
Stool from human immunodeficiency virus (HIV)–exposed, uninfected infants randomized to receive or not receive cotrimoxazole prophylaxis was collected, sequenced, and analyzed. *A*, HIV-positive mothers with HIV-uninfected infants were enrolled in the study. Their HIV-negative infants were randomized to either receive cotrimoxazole prophylaxis according to World Health Organization guidelines (red, CTX-T) or to not receive cotrimoxazole prophylaxis (blue, CTX-N). *B*, Three stool samples were collected per infant at 6 weeks (timepoint A), 4 months (timepoint B), and 6 months (timepoint C). Spread in collection time is due to variability in patient visit time. *C*, Infant stool samples were then shotgun whole-metagenome sequenced.

To address concerns about effects of cotrimoxazole prophylaxis on developing HEU infant gut microbiota, we sequenced 163 stool samples from a randomized controlled trial of cotrimoxazole treatment in 63 HEU infants [26]. Stool collections were concentrated during the first 6 months of life, referred to as the first stage of microbiome development [18]. Several studies suggest that infants are most vulnerable to gut microbiome perturbations during this period [18, 23]. We analyzed HEU infant stool metagenomes to determine their gut microbial taxonomic composition, microbiome functional genes, and antibiotic resistomes (Figure 1). We then compared these microbiome characteristics between cotrimoxazole-treated HEU infants (CTX-T infants) and HEU infants not treated with cotrimoxazole (CTX-N infants).

## MATERIALS AND METHODS

### Sample and Metadata Collection

Ethical approval was obtained from the Biomedical Research Ethics Committee, University of KwaZulu-Natal, South Africa (BFC212/13). Before enrollment, written informed consent was obtained from mothers visiting the Cato Manor and Lancers Road Clinics in Durban, South Africa, for routine clinic appointments. Infants with birthweights >2000 g born to HIV-infected mothers were included and followed for 1 year. Infants and their mothers had to be receiving antiretroviral prophylaxis prior to enrollment. Infants had to have a negative HIV DNA polymerase chain reaction result by COBAS AmpliPrep/COBAS TaqMan HIV-1 qualitative test prior to enrollment and their first sample collection timepoint at approximately 6 weeks of age. Infants were excluded if they had any illnesses, antibiotics (including cotrimoxazole), or traditional medicines, and all infants were exclusively breastfed. HEUs were randomized at their first timepoint (6-week) clinic visit to receive or not receive cotrimoxazole therapy. Cotrimoxazole was administered once daily during the study period; infants <5 kg received 20 mg trimethoprim/100 mg sulfamethoxazole orally and infants 5–15 kg received 40 mg trimethoprim/200 mg sulfamethoxazole orally. Stool samples were collected from infants at 3 timepoints (6 weeks, 4 months, and 6 months). Fresh stool from plastic-lined diapers was transferred to 2 mL BD cryovials using disposable wooden sticks. Samples were frozen within 6–8 hours of collection and stored at −80°C until analysis as described previously by Dan et al and Agapova et al [27, 28]. Two hundred thirteen stool specimens were collected from 70 infants during the study. Infants with an initial 6-week stool and at least 1 subsequent sample (63 infants) were shotgun-sequenced and included.

### Metagenomic DNA Extraction

PowerSoil DNA Isolation Kit (MoBio Laboratories) was used to extract metagenomic DNA from approximately 150 mg of each stool sample. Manufacturer protocol was used with minor modifications for sample lysis: 2 rounds of 2 minutes of bead beating at 2.5 k oscillations per minute for 2 minutes followed by 1 minute on ice and 2 additional minutes of beadbeating using a Mini-Beadbeater 24 (Biospec Products). DNA was quantified using a Qubit fluorometer dsDNA HS Assay (Invitrogen) and stored at −20°C.

### Metagenomic Sequencing Library Preparation

Metagenomic DNA was diluted to 0.5 ng/µL before sequencing library preparation. Sequencing libraries were prepared with a Nextera DNA Library Prep Kit (Illumina) as described in Baym et al [29]. Libraries were purified using the Agencourt AMPure XP system (Beckman Coulter) and quantified using the Quant-iT PicoGreen dsDNA assay (Invitrogen). For each sequencing lane, 10 nM of approximately 96 samples was pooled 3 independent times. These pools were quantified using the Qubit dsDNA BR Assay and combined in an equimolar fashion. Samples were submitted for 2 × 150-bp paired-end sequencing on an Illumina NextSeq High-Output platform with a target sequencing depth of 3 million reads per sample.

### Metagenomic Profiling

Illumina paired-end reads were binned by index sequence. Adapter and index sequences were trimmed and sequences were quality-filtered using Trimmomatic version 0.38 [30] with these parameters: *java -Xms2048m -Xmx2048m -jar trimmomatic-0.38.jar PE -phred33 ILLUMINACLIP: NexteraPE-PE.fa:2:30:10:1:TRUE SLIDINGWINDOW:4:15 LEADING:10 TRAILING:10 HEADCROP:15 MINLEN:60*. Microbial taxa relative abundance was calculated using MetaPhlAn2 [31] (repository tag 2.6.0). Metabolic pathway abundance was determined using HUMAnN2 [32] (repository tag 0.11.2). Raw count values were normalized for sequencing depth and collapsed by ontology, and tables were merged using HUMAnN2 utility scripts [32].

Resistance gene abundance including for *dfr*/*sul* genes was calculated using ShortBRED [33]. Marker sequences were built using the Comprehensive Antibiotic Resistance Database [34] with *shortedbread_identify.py*. Default parameters were used with exception for *-clustid* 0.95. Uniref90 [35] was the reference masking protein database. Efflux pumps and regulatory subunits were removed due to their lower specificity to antimicrobial resistance compared to other genes in the database.

### Statistical Analysis

Where possible, day of life rather than timepoint was used to account for variation in patient visit dates. Statistical analysis was conducted in R software version 3.5.3 [36]. Visualizations were made using ggplot2 version 3.1.0 [37], ggpubr version 0.2 [38], and cowplot version 0.9.4 [39]. The vegan: Community Ecology Package version 2.5–4 [40] was used for canonical analysis of principal coordinates [41] (*capscale* function), α- and β-diversity calculations, and permutational multivariate analysis of variance tests (*adonis2* function). Linear mixed-effects models were implemented with lme4 version 1.1–21 (*lmer* function) [42]. Dabestr version 0.2.0 [43] was used for bootstrapping samples and for calculating confidence intervals (CIs). Stats (base R) version 3.5.3 was used for statistical calculations. The *wilcox.test* function was applied with *paired = T/F* depending on if comparisons were inside or outside of the treatment group. The *fisher.test* function was for clinical metadata comparisons. The *p.adjust* function was applied where appropriate to correct for multiple hypothesis testing with *method=“fdr”* (Benjamini-Hochberg [44]). Log-transformation was implemented using the *log* function with default parameters.

### Data Availability

Shotgun metagenomic reads (quality-checked and filtered for human reads) generated for this study were uploaded to the National Center for Biotechnology Information (NCBI) under the project code PRJNA549787 (see BioSample accession metadata file for individual sample accession codes). Metadata files for these shotgun metagenomic reads are also included in this submission: (1) SACTX_HEU_Infant_metadata.txt; (2) SACTX_DOL_metadata_mod.txt; (3) SACTX_HEU_Infant_bodyMeasurements.txt; (4) SACTX_HEU_Infant_Bloodwork.txt; (5) NCBI_BioSample_AccessionList.txt.

## RESULTS

### Maternal CD4 Count, Infant Physical Traits, Biological Markers, and Reported Illnesses Were Not Different Between CTX-T and CTX-N Infants

**Figure 2.**
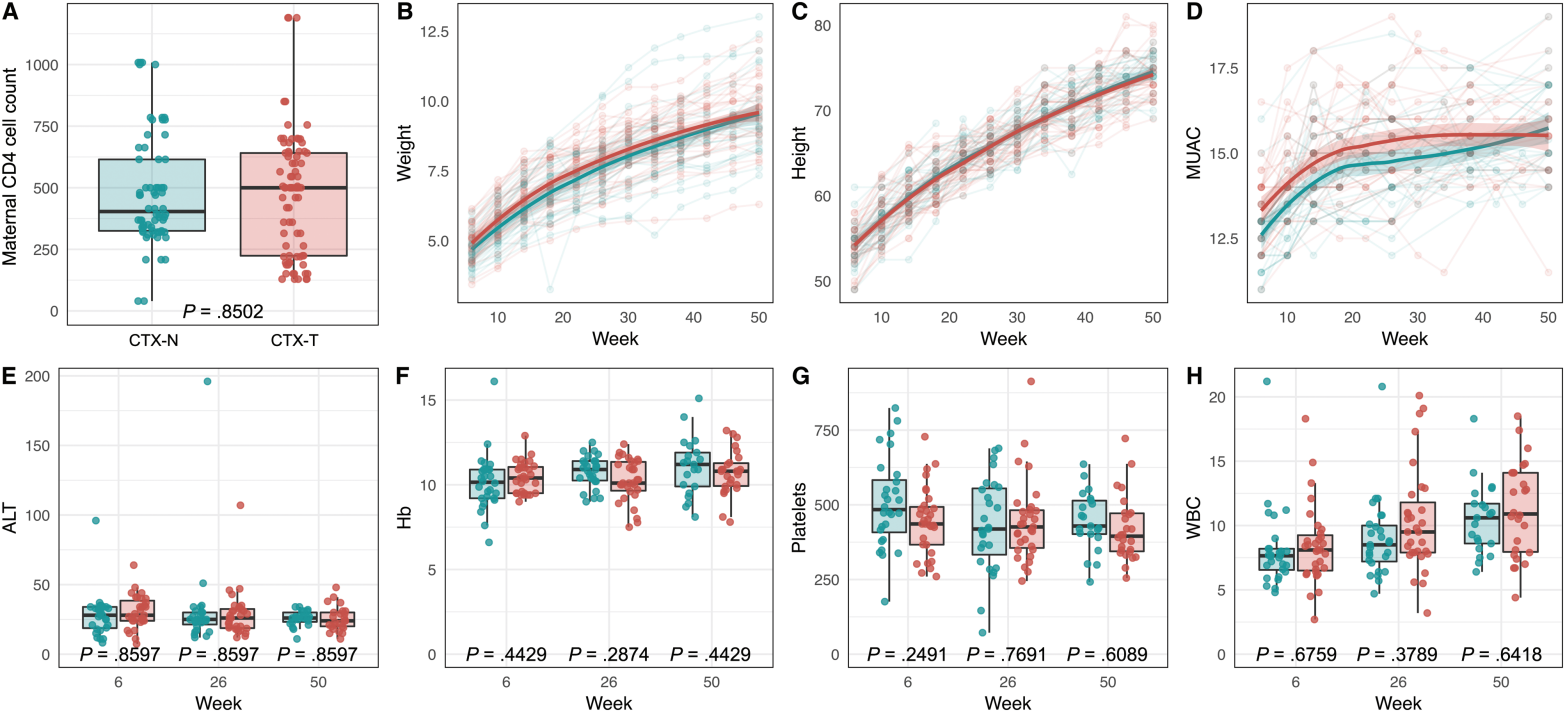
Maternal CD4 cell count, physical traits, and blood testing results are not different between cotrimoxazole-treated human immunodeficiency virus–exposed, uninfected (HEU) infants (CTX-T; red) and HEU infants not treated with cotrimoxazole (CTX-N; blue). Points represent individual measurements for infants. *A*, Comparison (unpaired Wilcoxon rank-sum test) of maternal CD4 count between CTX-T infants and CTX-N infants. Loess regression (bold line) with 95% confidence interval (shaded area) for CTX-T infants and CTX-N infants for weight (*B*), height (*C*), and mid-upper arm circumference (MUAC) (*D*). Boxplots (*E–H*) show median values (dark middle line) and first and third quartiles (lower and upper lines). Between-group comparisons for *E–H* are made using unpaired Wilcoxon tests (rank-sum) with Benjamini-Hochberg correction. Comparison between CTX-T infants and CTX-N infants at 3 separate collection times for alanine aminotransferase (ALT) (*E*), hemoglobin (Hb) (*F*), platelets (*G*), and white blood cell (WBC) count (*H*).

Maternal CD4 T-cell counts, anthropometric measurements (height, weight, and mid-upper arm circumference), biological markers (alanine aminotransferase, hemoglobin, platelets, and white blood cell count), and reported illnesses did not differ by cotrimoxazole treatment (Figure 2, Supplementary Notes E, and Supplementary Figure 1). As these metrics were indistinguishable between CTX-T and CTX-N infants, we investigated microbiome characteristics for intergroup differences.

### CTX-T Infants and CTX-N Infants Separated by Resistance Gene Content Following Cotrimoxazole Treatment

**Figure 3.**
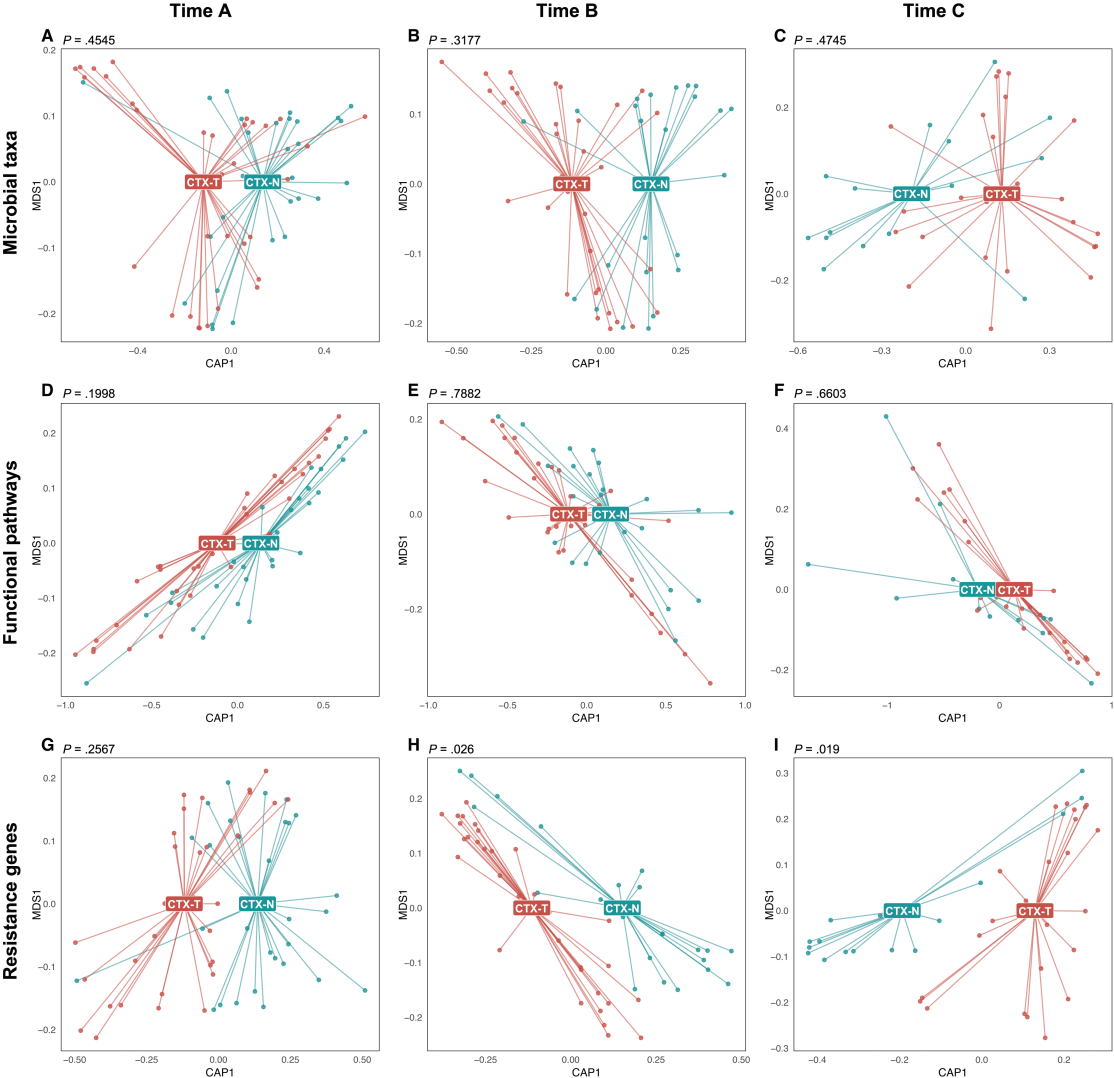
Cotrimoxazole-treated human immunodeficiency virus–exposed, uninfected (HEU) infants (CTX-T; red) and HEU infants not treated with cotrimoxazole (CTX-N; blue) significantly separate by resistance gene annotation profiles, but not by microbial taxonomic or functional pathway annotation profiles. Points are individual samples and labels are centers of gravity for CTX-T infants and CTX-N infants from canonical analysis of principal coordinates on dissimilarity matrices calculated by Bray-Curtis. Permutational multivariate analysis of variance was used to determine if group separations were significant, and *P* values are reported above each plot. Input data was generated by MetaPhlAn2 (microbial taxonomic profiles) for *A–C*, by HUMAnN2 (functional pathway annotations) for *D–F*, and by ShortBRED (resistance gene annotations) for *G–I*. Abbreviations: CAP1, Canonical Analysis of Principal coordinates; MDS1, multidimensional scaling.

While microbial taxa and functional pathways did not have significantly detectable differences (Figure 3A–F), resistance gene differences between CTX-T and CTX-N infants manifested in timepoints B and C (Figure 3H and 3I; *P* = .026 and *P* = .019). This suggests that cotrimoxazole treatment in HEU infants affects resistance genes more than microbial taxonomic composition or functional metabolic pathways. Importantly, this separation manifested at 4 months (timepoint B) and 6 months (timepoint C), but it was not present at 6 weeks (timepoint A) before treatment began.

### CTX-T Infants Had Higher Resistance Gene Abundance Than CTX-N Infants Following Cotrimoxazole Treatment

**Figure 4.**
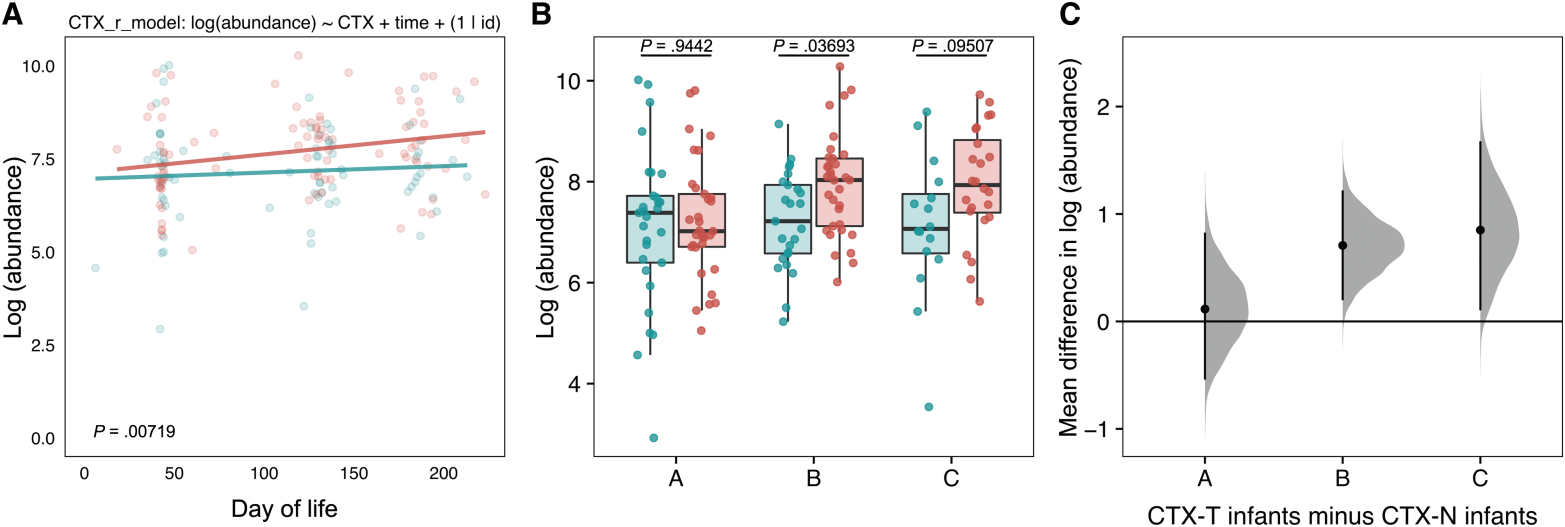
Total resistance gene abundance is higher in cotrimoxazole-treated human immunodeficiency virus–exposed, uninfected (HEU) infants (CTX-T) than in HEU infants not treated with cotrimoxazole (CTX-N). *A*, Points represent individual HEU infant samples colored by treatment group (red for CTX-T infants and blue for CTX-N infants), and lines represent predictions of simple linear regression models for the 2 groups. The x-axis for each plot is the day of life for each infant calculated from their day of birth, and the y-axis is log-transformed total resistance gene abundance. The formula for the linear mixed-effects model is reported above the plot. The model was compared to a null model without the cotrimoxazole treatment variable (CTX) included using a likelihood ratio test. The *P* value for this comparison is reported at bottom left. *B*, Points are individual HEU infant samples binned by treatment group (red for CTX-T and blue for CTX-N) and by time. The y-axis is log-transformed total resistance gene abundance. Unpaired Wilcoxon tests were used to compare the treated to untreated samples within collection times. *C*, Gray distributions show the difference at each collection time between the CTX-T infants and CTX-N infants log-transformed total resistance gene abundance for 5000 bootstrapped subsamples. The black points are the mean value for this distribution, and black lines are 95% confidence intervals.

To understand drivers of group separation for resistance genes in timepoints B and C (Figure 3H and 3I), we first looked at resistance gene abundance. Using a linear mixed-effects model with resistance gene abundance as the response variable, we found that cotrimoxazole treatment was a significant predictor of increased resistance gene prevalence (Figure 4A; *P* = .00719). Additionally, comparisons of CTX-T infants and CTX-N infants binned by timepoint (Figure 4B and 4C) demonstrated increased resistance gene prevalence measured by log-normalized resistance gene abundance in timepoint B (mean, 0.71 [95% CI, .2–1.2]) and timepoint C (mean, 0.85 [95% CI, .1–1.7]) in the CTX-T infants. The timepoint C comparison was not significant by false discovery rate–corrected Wilcoxon testing, but it was significant when tested by bootstrapping to calculate a 95% CI.

### Microbial Taxonomic and Resistance Gene α-Diversity Increased Significantly in CTX-T Infants Following Cotrimoxazole Exposure

**Figure 5.**
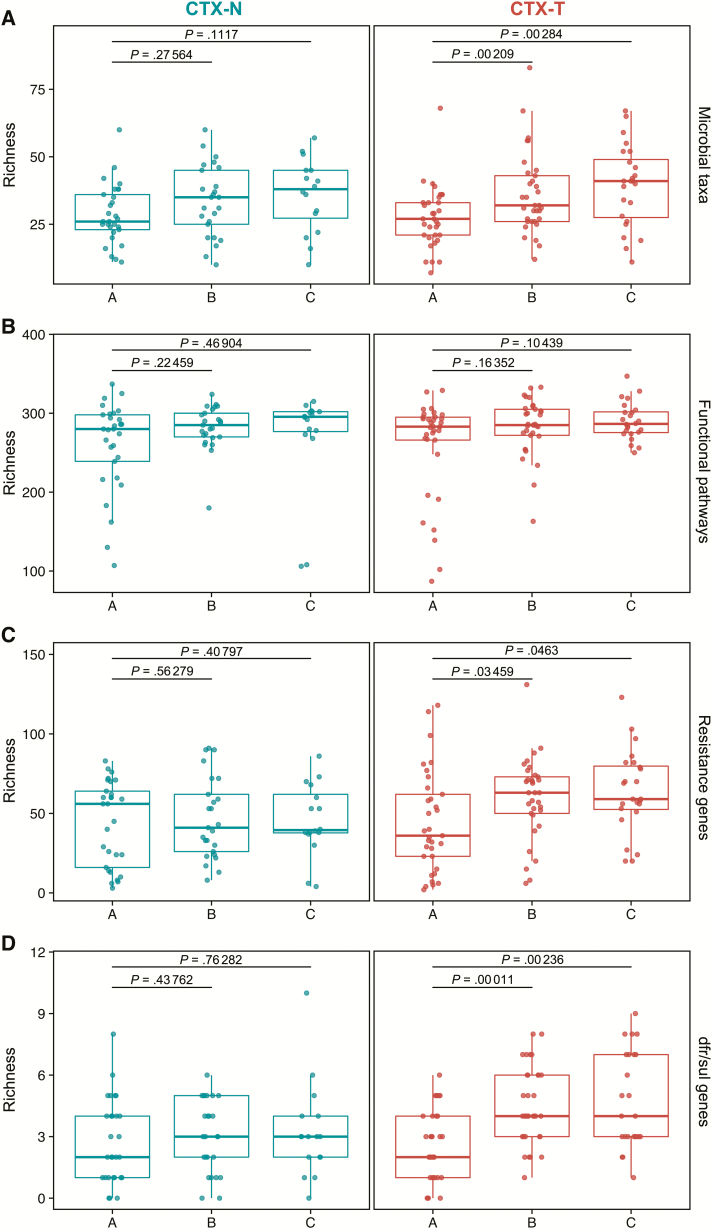
Resistance richness (α-diversity) significantly increases over time in cotrimoxazole-treated human immunodeficiency virus–exposed, uninfected (HEU) infants (CTX-T) for total resistance genes and for *dfr/sul* resistance genes. Points represent richness values for individual samples, and boxes show median values (dark middle line) and first and third quartiles (lower and upper lines). The x-axis groups for each plot are the times of collection, and the y-axis for each plot is richness. Paired Wilcoxon tests (signed rank) were used to compare the latter 2 collections (timepoints B and C) to the first collection (timepoint A), and *P* values are reported above the graph with black lines depicting the comparisons. Graphs show HEU infants not treated with cotrimoxazole (CTX-N; blue) on the left and CTX-T infants (red) on the right. Richness was calculated for microbial taxa (*A*), functional pathways (*B*), resistance genes (*C*), and trimethoprim- and sulfonamide-resistance (*dfr*/*sul*) genes (*D*).

Next, we investigated infant gut microbial taxonomic, functional metabolic pathway, and resistance gene α-diversity longitudinally in CTX-T infants and CTX-N infants (Figure 5A–C). The α-diversity is diversity in an area independent of other areas (in this study, the area is within each infant’s gut). We consistently observed that microbial taxonomic, functional metabolic pathway, and resistance gene α-diversity did not significantly increase from timepoint A to timepoints B or C for CTX-N infants. In contrast, CTX-T infants showed significant increases in α-diversity over time measured by richness (number of unique values) for microbial taxonomic profiles (Figure 5A; timepoint A to timepoint B: *P* = .0021; timepoint A to timepoint C: *P* = .0028) and resistance genes (Figure 5C; timepoint A to timepoint B: *P* = .034; timepoint A to timepoint C: *P* = .046). Shannon index α-diversity measurements yielded similar results (Supplementary Notes A and Supplementary Figure 2). Importantly, maternal CD4 T-cell count and reported illnesses did not significantly alter HEU infant gut microbial taxa α-diversity (Supplementary Notes D and Supplementary Figures 3–5).

Clinical resistance to trimethoprim is often mediated by transferrable *dfr* genes, and clinical resistance to sulfamethoxazole is often mediated by transferrable *sul* genes [10]. We observed significant increases in α-diversity of cotrimoxazole-specific *dfr* and *sul* resistance genes for CTX-T infants relative to the preintervention timepoint (Figure 5D; timepoint A to timepoint B: *P* = .00011; timepoint A to timepoint C: *P* = .00236).

### Cotrimoxazole Treatment Resulted in Significantly Higher Resistance Gene α-Diversity for CTX-T Infants Compared to CTX-N Infants

**Figure 6.**
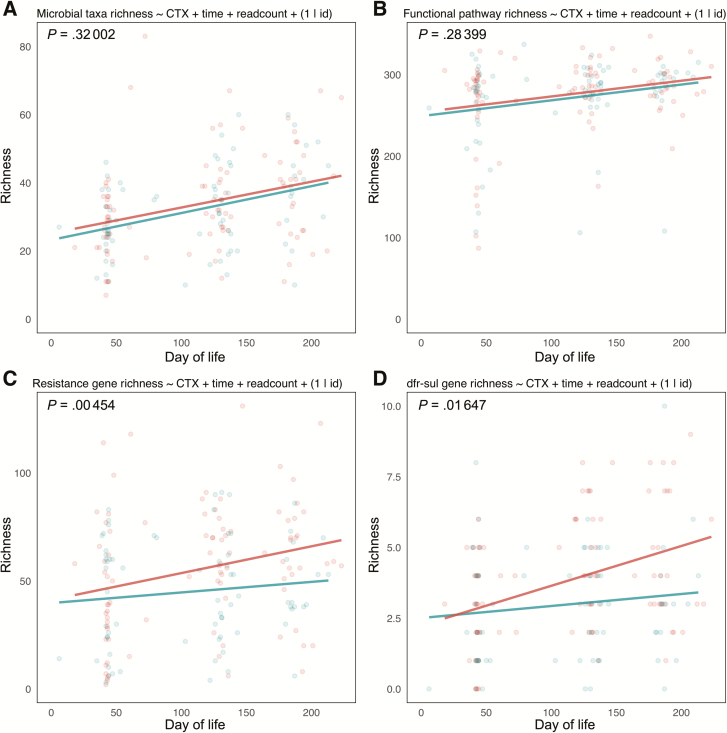
Resistance gene richness (α-diversity) is significantly higher in cotrimoxazole-treated human immunodeficiency virus–exposed, uninfected (HEU) infants (CTX-T) compared to HEU infants not treated with cotrimoxazole (CTX-N). Points represent individual patient samples colored by treatment group (red for CTX-T infants and blue for CTX-N infants), and lines represent predictions of simple linear regression models for the 2 groups. The x-axis for each plot is the day of life for each infant calculated from their day of birth, and the y-axis is richness. Models were made for microbial taxa (*A*), functional pathways (*B*), resistance genes (*C*), and trimethoprim- and sulfonamide-resistance (*dfr*/*sul*) genes (*D*). Formulas for each linear mixed-effects model are reported above the plots, and these models were compared using likelihood-ratio tests to null models made without the cotrimoxazole treatment variable (CTX) included. The *P* values for these comparisons of linear mixed-effects models are reported at top left of each graph.

To determine if within treatment group differences were significant between treatment groups, we constructed linear mixed-effects models with α-diversity as the response variable and treatment, day of life, and sample read count as fixed-effects variables. We also included subject identity as a random-effects variable to account for repeated measures (Figure 6 and Supplementary Figure 6). When compared to null models where cotrimoxazole treatment was not included as a fixed effect, cotrimoxazole treatment significantly differentiated resistance gene richness (Figure 6C; *P* = .0045) and *dfr*/*sul* gene richness (Figure 6D; *P* = .016), but it did not have a significant effect on microbial taxa richness (Figure 6A) or functional pathway richness (Figure 6B).

### Cotrimoxazole Treatment Resulted in Lower Microbial Taxonomic and Resistance Gene β-Diversity for CTX-T Infants Compared to CTX-N Infants

**Figure 7.**
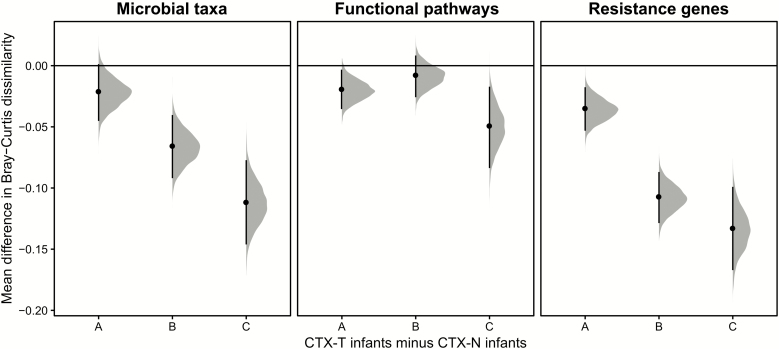
Microbial taxa, functional pathway, and resistance gene β-diversity are lower in cotrimoxazole-treated human immunodeficiency virus–exposed, uninfected (HEU) infants (CTX-T) than in HEU infants not treated with cotrimoxazole (CTX-N). Data are shown for β-diversity calculated from microbial taxonomic profiles, functional metabolic pathways, and resistance genes. Distributions show the difference at each collection timepoint between the CTX-T cohort’s Bray-Curtis dissimilarities and the CTX-N cohort’s Bray-Curtis dissimilarities for 5000 bootstrapped subsamples. The black points are the mean value for this distribution, and black lines are 95% confidence intervals.

We next compared microbial taxa, functional pathway, and resistance gene β-diversities of CTX-T infants and CTX-N infants within our 3 collection timepoints (Figure 7 and Supplementary Figure 7). We calculated pairwise Bray-Curtis dissimilarities [45] for our samples’ microbial taxonomic profiles, functional metabolic pathways, and resistance genes. β-diversity is diversity between 2 samples, and higher diversity indicates that sample compositions are more different from each other.

Within-treatment group comparisons showed sustained decreases for CTX-T infants in microbial taxa, functional metabolic pathway, and resistance gene β-diversity from 6 weeks to 4 months and to 6 months (Supplementary Notes C and Supplementary Figure 8).

In all cases, CTX-T infants had lower microbial taxonomic, functional pathway, and resistance gene β-diversity than CTX-N infants (Figure 7 and Supplementary Figure 7), indicating that microbiome characteristics in CTX-T infants became more similar to one another after starting cotrimoxazole treatment. This dissimilarity was significantly lower in CTX-T infants compared to CTX-N infants for microbial taxa at timepoint B (mean, −0.067 [95% CI, −.092 to −.04]) and timepoint C (mean, −0.11 [95% CI, −.15 to −.077]). The magnitude of difference between CTX-T and CTX-N infants also increased with time. Interestingly, functional pathways were significantly different between CTX-T infants and CTX-N infants at timepoint A (mean, −0.020 [95% CI, −.035 to −.0033]) and timepoint C (mean, −0.050 [95% CI, −.084 to −.017]) but not at timepoint B (mean, −0.0088 [95% CI, −.026 to .0083]). Resistance gene β-diversity was significantly lower in timepoint A (mean, −0.036 [95% CI, −.053 to −.018]), timepoint B (mean, −0.11 [95% CI, −.13 to −.087]), and timepoint C (mean, −0.13 [95% CI, −.17 to −.099]) in CTX-T infants compared with CTX-N infants. Resistance gene differences between timepoint A and timepoints B and C were also significant, with 95% CIs not overlapping between early and later timepoints for the difference between CTX-T infants and CTX-N infants.

## DISCUSSION

Here we investigate the effects of cotrimoxazole prophylaxis on developing HEU infant gut microbiomes, finding that cotrimoxazole treatment has little effect on taxonomic or functional metabolic pathway α-diversity, but increases resistance gene α-diversity. While α-diversity increases or remains the same following cotrimoxazole treatment, taxonomic, functional metabolic pathway, and resistance gene β-diversity decreases.

Studies have noted that early-life microbiota perturbations have long-term health consequences [23–25, 46]. Perturbations can result from many factors, including HIV exposure in utero [47, 48] and antibiotic treatment [18]. In contrast to studies showing that microbiota taxonomic α-diversity decreases following antibiotic treatment [49–52], we did not find significant decreases in CTX-T infants compared to CTX-N infants, corresponding with our results for functional metabolic pathways and keystone taxa abundance (Supplementary Notes B). Gibson et al [21] showed that early-life microbiome α-diversity changes in preterm infants vary by antibiotic, so this may be specific for cotrimoxazole action on developing HEU infant gut microbiomes. Since cotrimoxazole is broad-spectrum [53], it may evenly reduce gut bacterial load, making its effects undetectable by measuring α-diversity or relative abundance. Indeed, 2 studies of HIV-infected populations found that cotrimoxazole had insignificant effect on gut microbial diversity, but differences in study design qualify comparisons to our study (see Supplementary Notes F) [12, 54]. Alternatively, if cotrimoxazole reduces α-diversity, its effects on HEU infant gut microbiomes may be insignificant compared to perturbation from HIV exposure in utero [47, 55]. This possibility is supported by small but statistically significant increases in taxonomic α-diversity from timepoint A to timepoints B and C in CTX-T infants but not in CTX-N infants. This increase in microbial taxa α-diversity may result from initial suppression by cotrimoxazole followed by colonization with cotrimoxazole resistant or tolerant species. Finally, cotrimoxazole resistance may be prevalent enough in our cohort’s early colonizers that cotrimoxazole treatment does not perturb the HEU infant gut microbial community. Studies showing high resistance rates in low- and middle-income countries, including South Africa [56, 57], support this possibility [58]. Together, our taxonomic and functional microbiome data do not indicate large or consistent HEU infant gut microbiome dysbiosis in response to cotrimoxazole treatment.

In contrast with results for taxonomic composition and functional metabolic pathways, cotrimoxazole treatment significantly changed HEU infant antibiotic resistomes. Changes manifested as increases in resistance gene prevalence and resistance gene α-diversity. Importantly, increases are found for trimethoprim and sulfamethoxazole–specific resistance genes and for resistance genes as a whole. This is consistent with results by Powis et al [59] showing that *Escherichia coli* and *Klebsiella pneumoniae* commensal bacteria isolated from CTX-T infants had increased cotrimoxazole and amoxicillin resistance.

Microbiota β-diversity decreases over time for infants in this cohort, but in CTX-T infants, β-diversity reductions are swifter and more pronounced than in CTX-N infants. This could be from selective pressure of cotrimoxazole treatment on specific bacterial taxa. As functional metabolic pathways and resistance genes are tied to bacterial taxa, their β-diversity would also be sensitive to the selective pressure of antibiotics. Interestingly, sensitivity of microbial taxonomic profiles, functional metabolic pathways, and resistance genes to cotrimoxazole treatment differs. Resistance genes are most sensitive to antibiotic pressures and functional metabolic pathways are least sensitive. Functional metabolic pathways are likely conserved between multiple bacterial taxa, and this conservation would make them invariant to selective pressures [60–62].

Our study adds to growing work on HEU infants [13, 15, 47, 48, 59, 63] (see Supplementary Notes F). To our knowledge, this is the first randomized controlled trial comparing cotrimoxazole-treated and untreated HEU infant gut microbiomes. Despite our efforts, this study has limitations. Since our collection timepoints only extend approximately 6 months, further changes may manifest between CTX-T and CTX-N infants after our sampling. Alternatively, resistance gene prevalence and diversity differences may equalize between the 2 groups. Our collection times are staggered due to patient appointment availability, and this staggering could reduce power to detect microbiota differences. Second, we can only establish relative and not absolute abundance differences; thus, we cannot determine if CTX-T infants and CTX-N infants differ in total gut bacterial load. Additionally, we do not have maternal metagenomes, in utero data, or cytokine information. Prior research has shown maternal factors such as diet and antimicrobial use can impact infant microbiomes and inflammatory markers [64, 65]. Finally, adherence difficulties are mentioned in the WHO guidelines [9]. Given daily dosing regimens, we cannot verify treatment adherence beyond verbal confirmation from mothers that infants received cotrimoxazole and did not receive other antibiotics.

Our study sheds light on the effects of cotrimoxazole prophylaxis on HEU infant microbiomes and provides additional information for future treatment recommendations. Unlike some other studies on antibiotic use in infants [66–69], we did not observe significant microbiome dysbiosis. However, increases we observed in resistance gene prevalence and diversity are important for ongoing public health and antibiotic stewardship discussions [58]. WHO guidelines say that “understanding whether people living with HIV using cotrimoxazole affects community cotrimoxazole resistance and whether community cotrimoxazole resistance affects treatment failure for other infectious diseases is important for national efforts to combat antimicrobial resistance” [9]. Low- and middle-income countries, including South Africa, often have high background antibiotic resistance rates [58]. Unnecessary antibiotic prescriptions may contribute to resistance disparities, and there is mounting evidence that cotrimoxazole treatment of HEU infants in countries with low malaria prevalence is ineffective [13]. As South Africa has low malaria prevalence and high infant HIV exposure, WHO recommendations on prophylactic cotrimoxazole may unnecessarily contribute to increasing resistance; however, more research on treatment consequences of high background antibiotic resistance for HIV-exposed infants is needed to refine guidelines. Additionally, dried blood PCR testing takes time and money [70]; thus, cotrimoxazole treatment often begins before HIV status is known. Furthermore, HIV-exposed infants who are HIV-negative at birth may contract HIV during breastfeeding [71]. These factors must also be considered in WHO recommendations, and they highlight urgency to develop rapid and accessible technologies to determine HIV infection status.

## Supplementary Data

Supplementary materials are available at *Clinical Infectious Diseases* online. Consisting of data provided by the authors to benefit the reader, the posted materials are not copyedited and are the sole responsibility of the authors, so questions or comments should be addressed to the corresponding author.

ciz1186_suppl_Supplementary_Figures

ciz1186_suppl_Supplementary_Notes

## Notes

***Author contributions.*** E. M.-G. and A. C. conceived the clinical study design. A. W. D., G. D., I. T., and B. B. conceived the metagenomic study design. A. W. D., under advisement of G. D., conducted the analysis and drafted the manuscript. E. M.-G. led sample collection and tabulated clinical metadata with help from B. D., and B. B., B. W., and X. S. extracted and sequenced the stool samples. T. K. helped with data processing and analysis. All authors critically reviewed the manuscript and provided feedback.

***Acknowledgments.*** The authors thank the clinics for the allocated clinic space and their partnership in taking care of these mother–child pairs. The authors acknowledge all the mothers, their children, and the study team for their commitment to this investigation. The authors also thank members of the Dantas laboratory for helpful discussions of the results, and the Edison Family Center for Genome Sciences and Systems Biology staff, Eric Martin, Brian Koebbe, and Jessica Hoisington-López for technical support and sequencing expertise.

***Disclaimer.*** The findings and conclusions of this study are those of the authors and do not reflect official positions of the funding agencies.

***Financial support.*** This work is supported in part by awards to G. D. from the National Institute of General Medical Sciences (grant number R01 GM099538), the National Institute of Allergy and Infectious Diseases (grant number R01 AI123394), and the Eunice Kennedy Shriver National Institute of Child Health and Human Development (grant number R01 HD092414) of the National Institutes of Health; the Edward Mallinckrodt Jr Foundation (Scholar Award); and the Bill & Melinda Gates Foundation Grand Challenges (GCE phase I award number OPP1161117). A. W. D. received support from the Institutional Program Unifying Population and Laboratory-Based Sciences Burroughs Wellcome Fund grant to Washington University. E. M.-G. received support from the International Atomic Energy Agency international training fellowship and the University of KwaZulu-Natal postdoctoral scholarship.

***Potential conflicts of interest.*** The authors: No reported conflicts of interest. All authors have submitted the ICMJE Form for Disclosure of Potential Conflicts of Interest.
