# Supplementary material for: Cotrimoxazole Prophylaxis Increases Resistance Gene Prevalence and α-Diversity but Decreases β-Diversity in the Gut Microbiome of Human Immunodeficiency Virus–Exposed, Uninfected Infants
Source: Clin Infect Dis. 2019 Dec 13;71(11):2858–68. doi: 10.1093/cid/ciz1186 (PMC7778358; doi:10.1093/cid/ciz1186)
Supplement: ciz1186_suppl_Supplementary_Figures [file ciz1186_suppl_supplementary_figures.pdf]

Supplementary Figures

Cotrimoxazole prophylaxis increases resistance gene prevalence and α-diversity but decreases β-diversity in the gut microbiome of HIV-exposed, uninfected infants.

Authors: Alaric W. D'Souza[1], Eshia Moodley-Govender[2], Bertram Berla[1,3], Tejas Kelkar[1], Bin Wang[1], Xiaoqing Sun[1], Brodie Daniels[2], Anna Coutsoudis[2], Indi Trehan[4], Gautam Dantas[1,3,5,6,**]

Affiliations:

[1]The Edison Family Center for Genome Sciences and Systems Biology, Washington University in St. Louis School of Medicine, Saint Louis, MO, USA

[2]Department of Paediatrics and Child Health, University of KwaZulu-Natal, Durban, South Africa

[3]Department of Pathology and Immunology, Washington University in St. Louis School of Medicine, St. Louis, MO, USA

[4]Department of Pediatrics, Washington University in St Louis School of Medicine, St. Louis, MO, USA

[5]Department of Molecular Microbiology, Washington University in St. Louis School of Medicine, Saint Louis, MO, USA

[6]Department of Biomedical Engineering, Washington University in St Louis, Saint Louis, MO, USA

**To whom correspondence should be addressed: dantas@wustl.edu

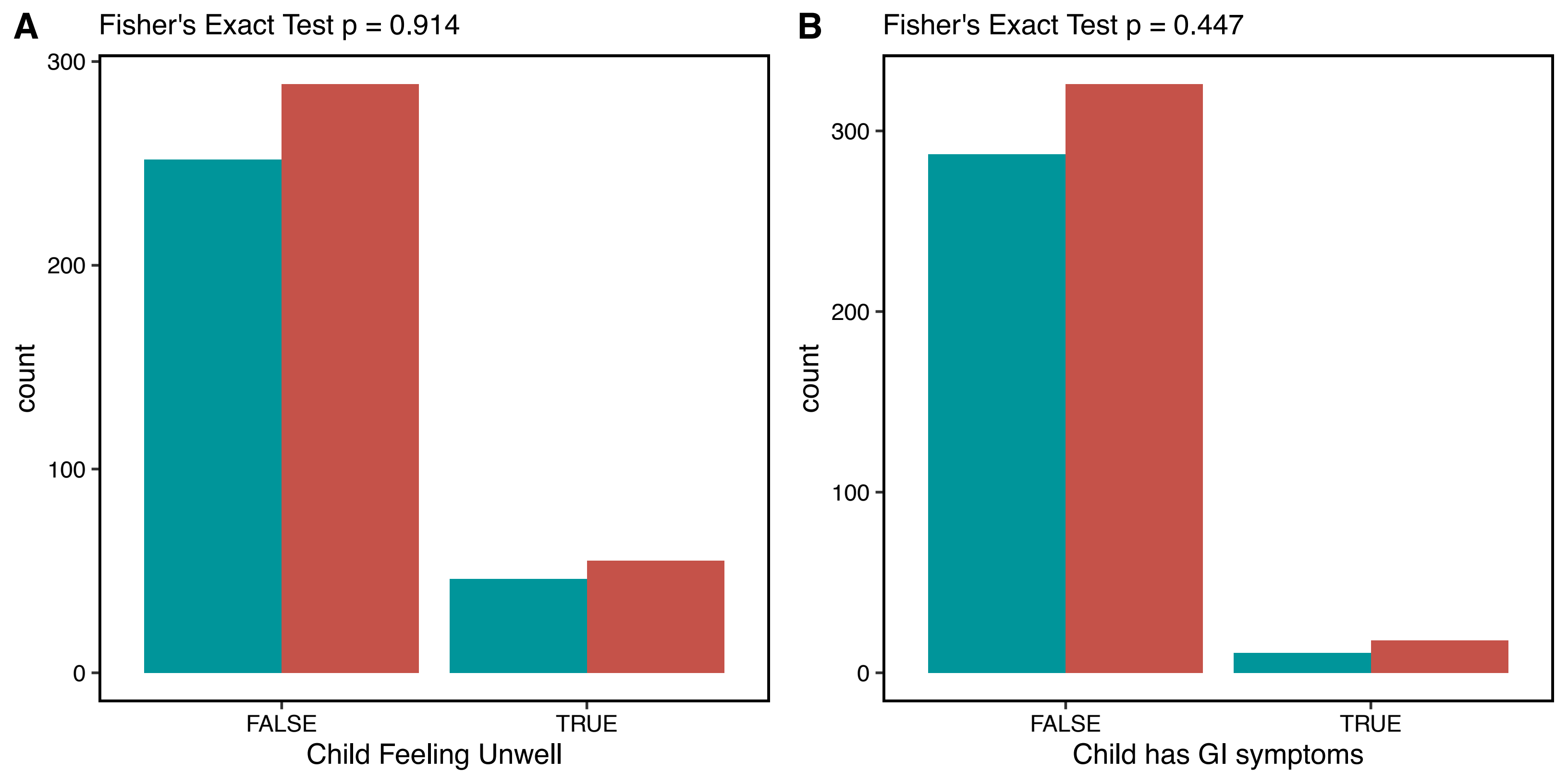
A
Fisher's Exact Test p = 0.914
300
200
100
0
count
FALSE
TRUE
Child Feeling Unwell
B
Fisher's Exact Test p = 0.447
300
200
100
0
count
FALSE
TRUE
Child has GI symptoms

Supplemental Figure 1: Reported illnesses did not differ significantly between CTX-T and CTX-N infants during the study period.

Comparison of reported **A.** all cause illness and **B.** gastrointestinal specific illness during the study period. TRUE indicates that an illness was reported and FALSE indicates no reported illness. Red bars correspond to counts for CTX-T infants and blue bars correspond to counts for CTX-N infants. Fisher's exact test was used to determine if either group was significantly more likely to report illness.

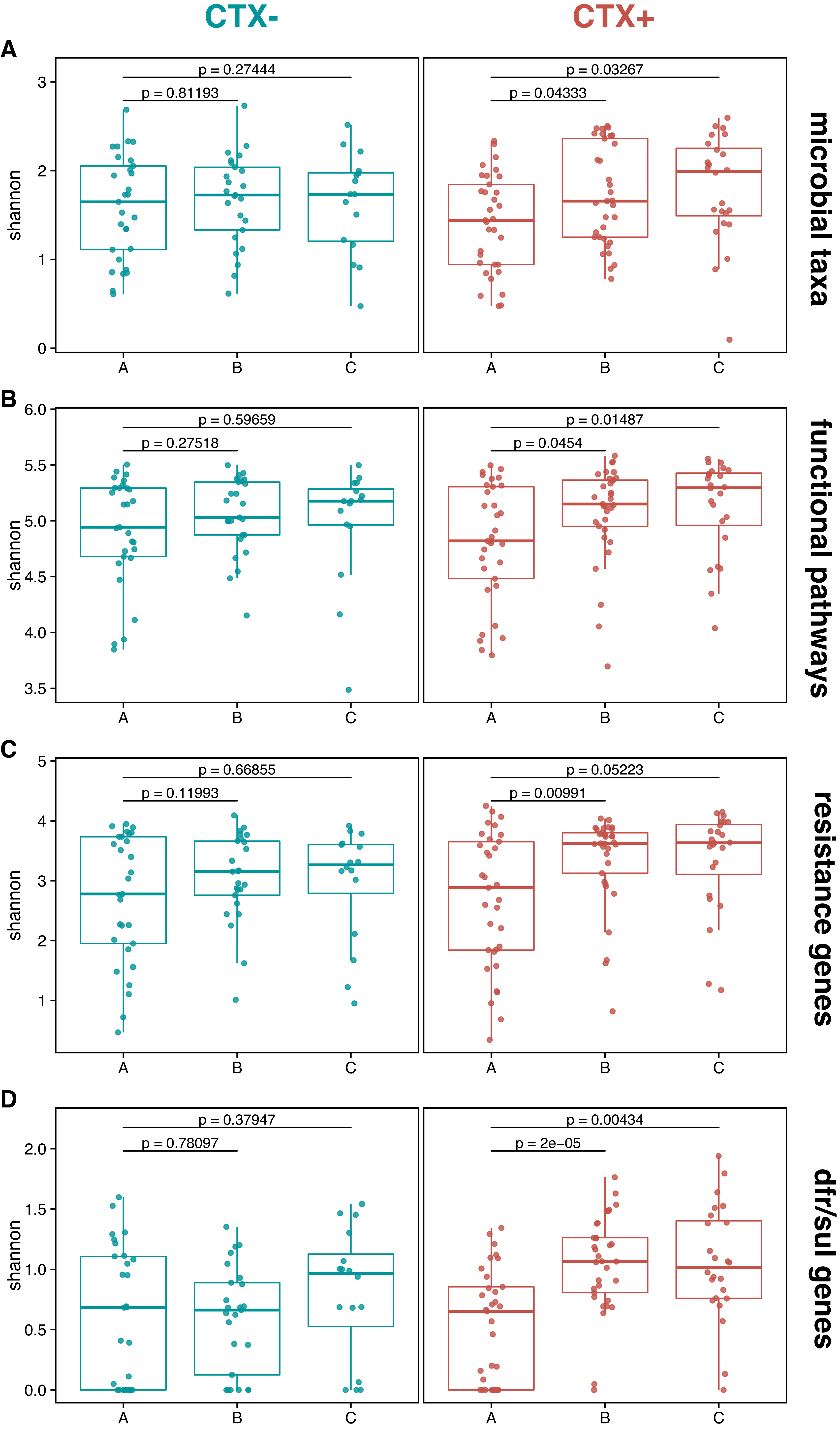

CTX-
CTX+
A
B
C
D
shannon
microbial taxa
functional pathways
resistance genes
dfr/sul genes
p = 0.27444
p = 0.81193
p = 0.03267
p = 0.04333
p = 0.59659
p = 0.27518
p = 0.01487
p = 0.0454
p = 0.66855
p = 0.11993
p = 0.05223
p = 0.00991
p = 0.37947
p = 0.78097
p = 0.00434
p = 2e−05

Supplemental Figure 2: Resistance gene Shannon Diversity (α-diversity) increases over time in CTX-T infants, but is stable for CTX-N infants

Points represent Shannon diversity values for individual samples and boxes show median values (dark middle line) and 1st and 3rd quartiles (lower and upper lines). x-axis groups for each plot are the times of collection and y-axis for each plot is richness. Paired Wilcoxon tests (signed rank) were used to compare the latter two collections (timepoints B and C) to the first collection (timepoint A) and p-values are reported above the graph with black lines depicting the comparisons. Graphs on the left show CTX-N infants (blue, CTX-) and on the right show CTX-T infants (red, CTX+). Shannon diversity was calculated for **A.** microbial taxa, **B.** functional pathways, **C.** resistance genes, and **D.** *dfr*/*sul* genes.

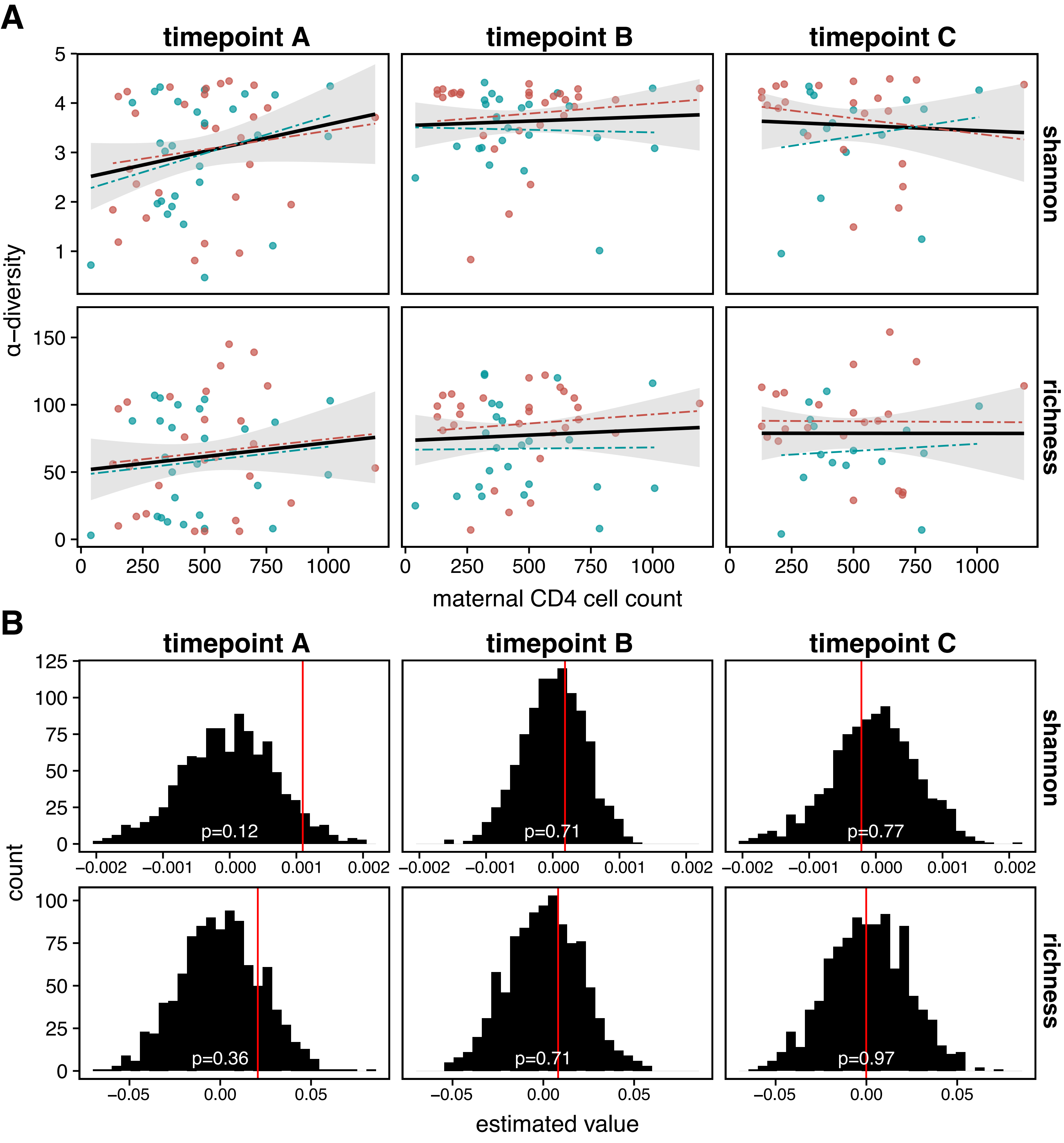
A
B
timepoint A
timepoint B
timepoint C
shannon
richness
α−diversity
maternal CD4 cell count
count
estimated value
p=0.12
p=0.71
p=0.77
p=0.36
p=0.71
p=0.97

Supplemental Figure 3: Maternal CD4 cell count does not significantly change microbiota α-diversity

A. Relationship between maternal CD4 count and HEU infant microbial taxa α-diversity. Microbial taxa α-diversity is on the y-axis and maternal CD4 count in on the x-axis. Maternal CD4 count was sampled once and is independent of infant sampling time separated by columns. Sample α-diversity is given for Shannon index above and richness below. Red dots correspond to CTX-T infants and blue dots are CTX-N infants. The black solid line is fitted to all points in the panel with the formula α-diversity ~ maternal CD4 count. Grey shaded area is the 95% CI for this line. Red and blue dashed lines are similarly fitted to CTX-T infants and CTX-N infants respectively. **B.** Expected (null) slope distribution from 1000 permutations compared to observed slope for relationship between maternal CD4 count and HEU infant microbial taxa α-diversity. Red vertical lines show the observed Estimate (slope) value for the panels in Supplemental Figure 3A and black histograms show the expected slope value under a null distribution based on linear models from 1000 permutations of the data in Supplemental Figure 3A. P-values calculated from the z-score are given in white for the deviation of the observed slope from the expected slope.

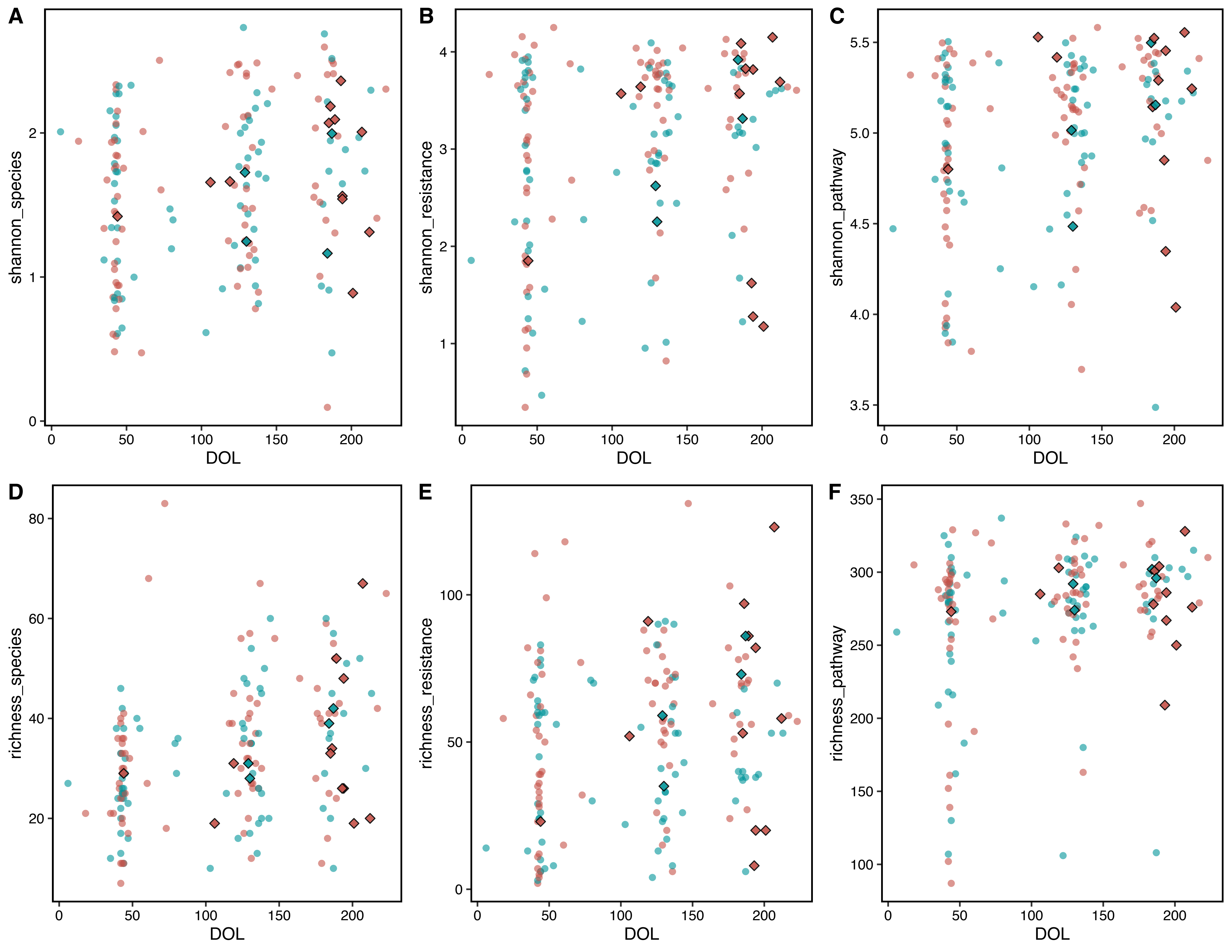

A
shannon_species
DOL
B
shannon_resistance
DOL
C
shannon_pathway
DOL
D
richness_species
DOL
E
richness_resistance
DOL
F
richness_pathway
DOL

Supplemental Figure 4: α-diversity does not vary by all cause reported illnesses. Points represent individual patient samples colored by treatment group (red for CTX-T infants and blue for CTX-N infants). Circular points are samples where no illness was reported, and diamond points with black outlines are samples where illness was reported. The x-axis for each plot is the day of life for each infant calculated from their day of birth. Y-axis is Shannon diversity for **A.** microbial taxa, **B.** resistance genes, **C.** functional pathways, and Richness for **D.** microbial taxa, **E.** resistance genes, **F.** functional pathways.

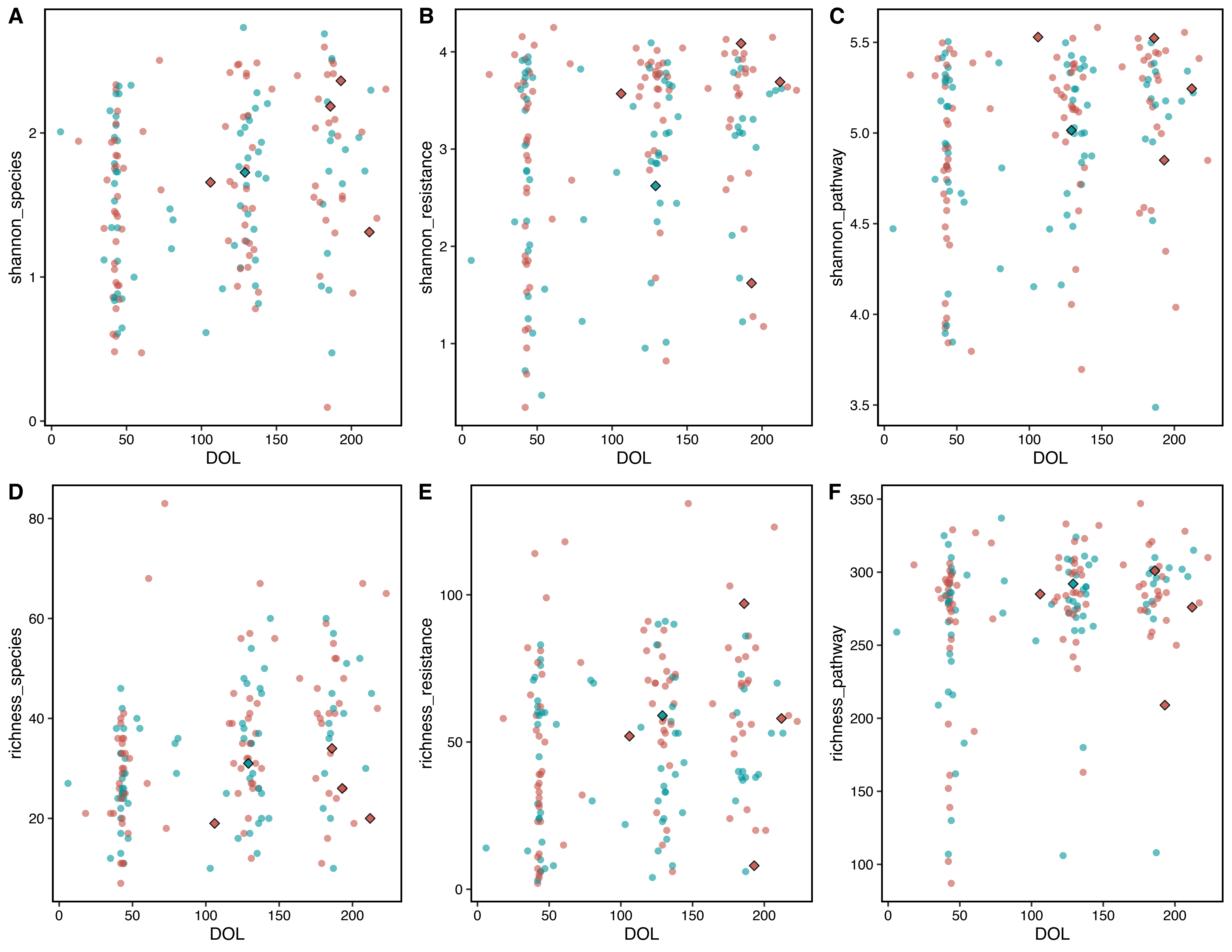
A
B
C
D
E
F
shannon_species
shannon_resistance
shannon_pathway
richness_species
richness_resistance
richness_pathway
DOL

Supplemental Figure 5: α-diversity does not vary by all cause reported gastrointestinal symptoms.

Points represent individual patient samples colored by treatment group (red for CTX-T infants and blue for CTX-N infants). Circular points are samples where no gastrointestinal illness (diarrhea or vomiting) was reported, and diamond points with black outlines are samples where gastrointestinal illness was reported. The x-axis for each plot is the day of life for each infant calculated from their day of birth. Y-axis is Shannon diversity for **A.** microbial taxa, **B.** resistance genes, **C.** functional pathways, and Richness for **D.** microbial taxa, **E.** resistance genes, **F.** functional pathways.

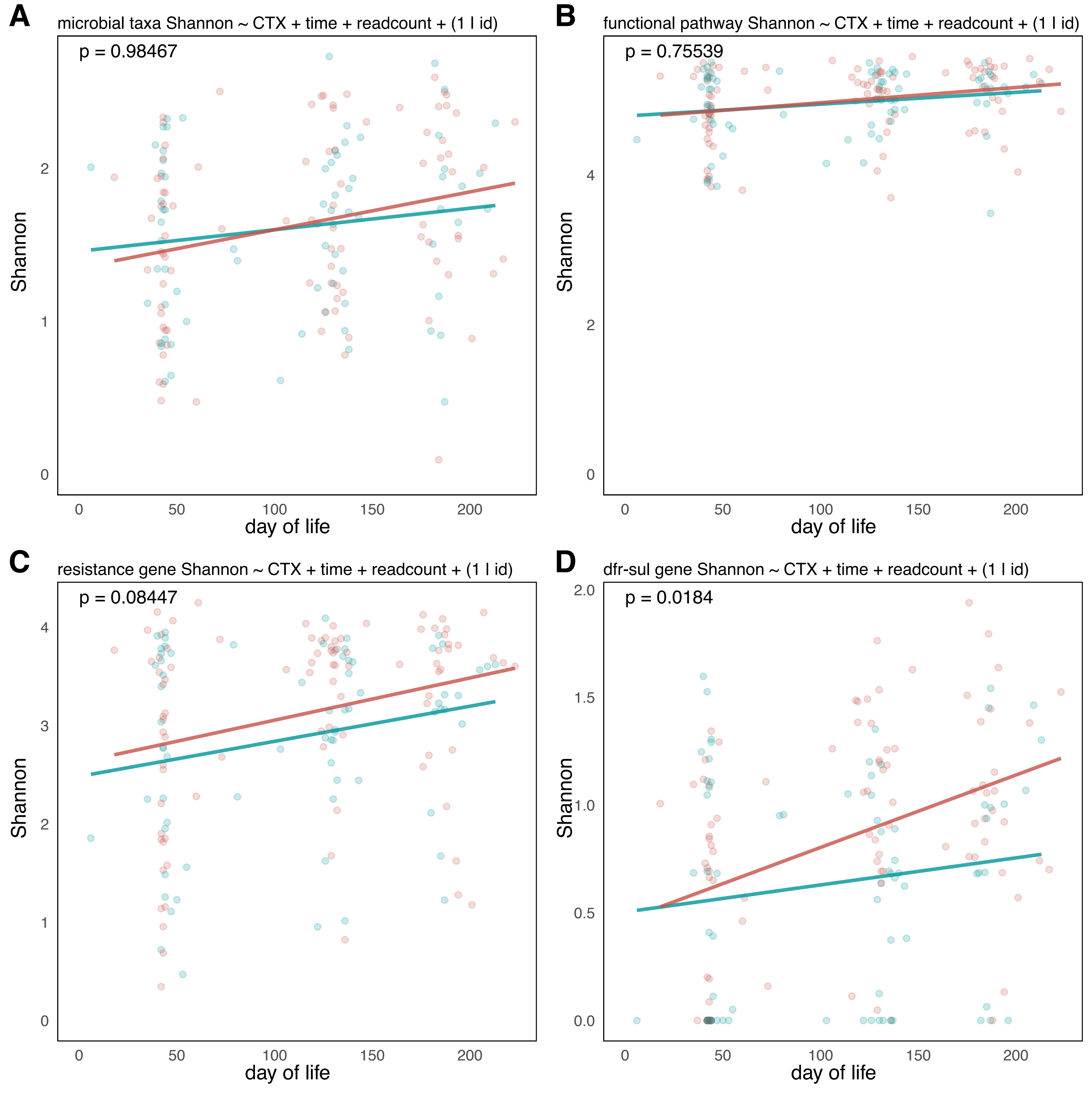

A
microbial taxa Shannon ~ CTX + time + readcount + (1 | id)
p = 0.98467
Shannon
day of life
B
functional pathway Shannon ~ CTX + time + readcount + (1 | id)
p = 0.75539
Shannon
day of life
C
resistance gene Shannon ~ CTX + time + readcount + (1 | id)
p = 0.08447
Shannon
day of life
D
dfr-sul gene Shannon ~ CTX + time + readcount + (1 | id)
p = 0.0184
Shannon
day of life

Supplemental Figure 6: *dfr/sul* gene Shannon diversity is significantly higher for CTX-T infants compared to CTX-N infants.

Points represent individual patient samples colored by treatment group (red for CTX-T infants and blue for CTX-N infants) and lines represent predictions of linear models for the two groups. The x-axis for each plot is the day of life for each infant calculated from their day of birth and y-axis is Shannon diversity. Models were made for **A.** microbial taxa, **B.** functional pathways, **C.** resistance genes, and **D.** trimethoprim- and sulphonamide-resistance (*dfr*/*sul*) genes. Formulas for each linear mixed-effects model are reported above the plots and these models were compared using likelihood-ratio tests to null models made without the cotrimoxazole treatment variable (CTX) included. The p-values for these comparisons of linear mixed-effects models are reported in the top left of each graph.

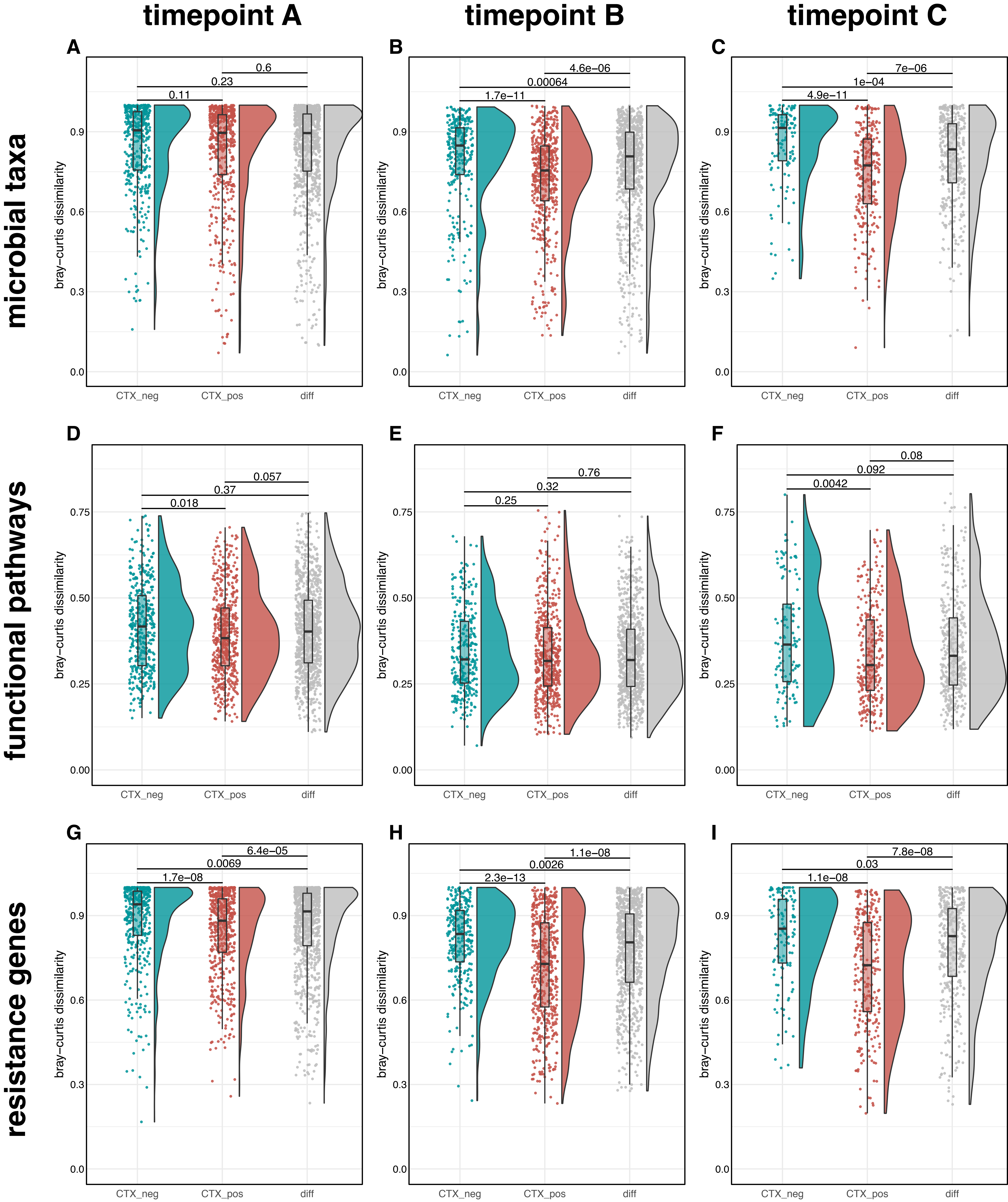

timepoint A
timepoint B
timepoint C
microbial taxa
functional pathways
resistance genes
A
B
C
D
E
F
G
H
I
bray−curtis dissimilarity
CTX_neg
CTX_pos
diff
0.6
0.23
0.11
4.6e−06
0.00064
1.7e−11
7e−06
1e−04
4.9e−11
0.057
0.37
0.018
0.76
0.32
0.25
0.08
0.092
0.0042
6.4e−05
0.0069
1.7e−08
1.1e−08
0.0026
2.3e−13
7.8e−08
0.03
1.1e−08

Supplemental Figure 7: Intragroup microbial taxonomic and resistance gene β-diversity for CTX-N infants is higher than CTX-T infant β-diversity and and intergroup β-diversity. Points represent Bray-Curtis dissimilarity between two samples (higher dissimilarity means samples were more different). Boxplots show median values (dark middle line) and 1st and 3rd quartiles (lower and upper lines). The distribution for each set of points is shown to the right of the points. Dissimilarities are blue if both compared samples are from CTX-N infants (CTX_neg), red if both samples are from CTX-T infants (CTX_pos), and grey if one sample is from a CTX-N infant and the other is from a CTX-T infant (diff). Dissimilarities were calculated from microbial taxa community matrices for **A**, **B**, and **C**, from functional pathway matrices for **D**, **E**, and **F**, and for resistance gene matrices for **G**, **H**, and **I**.

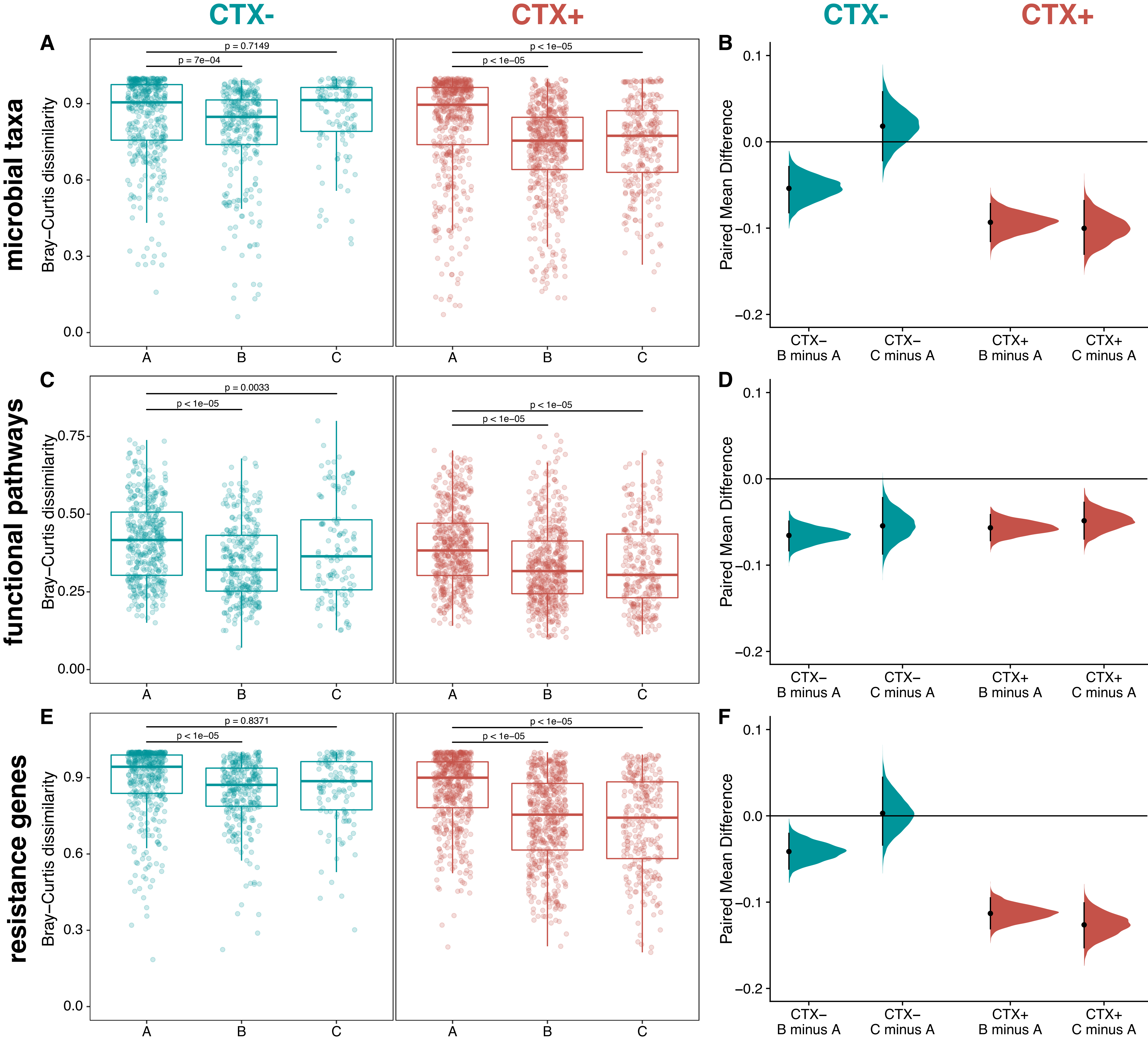
CTX-
CTX+
CTX-
CTX+
A
B
C
D
E
F
microbial taxa
functional pathways
resistance genes
Bray−Curtis dissimilarity
Paired Mean Difference
p = 0.7149
p = 7e−04
p < 1e−05
p = 0.0033
p = 0.8371
A
B
C
CTX−
B minus A
CTX−
C minus A
CTX+
B minus A
CTX+
C minus A

Supplemental Figure 8: Taxonomic, functional metabolic pathway, and resistance gene β-diversity decreases compared to baseline for CTX-T infants.

Boxplots with points show the pairwise Bray-Curtis dissimilarities for samples within time and treatment group (CTX-T infants in red and CTX-N infants in blue) for **A.** microbial taxonomic profiles, **C.** functional metabolic pathways, and **E.** resistance gene profiles. Comparisons are made to the initial time of collection (time A) using paired Wilcoxon tests and p-values are reported above the boxplots. Paired samplings were bootstrapped to get the distributions shown in **B.** for microbial taxonomic profiles, **D.** for functional metabolic pathways, and **F.** for resistance gene profiles. The distributions show deviation of times B and C from the initial time A for CTX-N infants (blue, CTX-) and CTX-T infants (red, CTX+). Black points represent the mean difference of the bootstrapped sampling distribution from the starting value in time A and the black lines represent 95% confidence intervals.
