# Supplementary material for: Cotrimoxazole Prophylaxis Increases Resistance Gene Prevalence and α-Diversity but Decreases β-Diversity in the Gut Microbiome of Human Immunodeficiency Virus–Exposed, Uninfected Infants
Source: Clin Infect Dis. 2019 Dec 13;71(11):2858–68. doi: 10.1093/cid/ciz1186 (PMC7778358; doi:10.1093/cid/ciz1186)
Supplement: ciz1186_suppl_Supplementary_Notes [file ciz1186_suppl_supplementary_notes.pdf]

Supplementary Notes

Cotrimoxazole prophylaxis increases resistance gene prevalence and α-diversity but decreases β-diversity in the gut microbiome of HIV-exposed, uninfected infants.

Authors: Alaric W. D'Souza[1], Eshia Moodley-Govender[2], Bertram Berla[1,3], Tejas Kelkar[1], Bin Wang[1], Xiaoqing Sun[1], Brodie Daniels[2], Anna Coutsoudis[2], Indi Trehan[4], Gautam Dantas[1,3,5,6,**]

Affiliations:

[1]The Edison Family Center for Genome Sciences and Systems Biology, Washington University in St. Louis School of Medicine, Saint Louis, MO, USA

[2]Department of Paediatrics and Child Health, University of KwaZulu-Natal, Durban, South Africa

[3]Department of Pathology and Immunology, Washington University in St. Louis School of Medicine, St. Louis, MO, USA

[4]Department of Pediatrics, Washington University in St Louis School of Medicine, St. Louis, MO, USA

[5]Department of Molecular Microbiology, Washington University in St. Louis School of Medicine, Saint Louis, MO, USA

[6]Department of Biomedical Engineering, Washington University in St Louis, Saint Louis, MO, USA

**To whom correspondence should be addressed: dantas@wustl.edu

Included in this Document

A. Shannon Index calculations for within group α-diversity

B. Linear-mixed effects model analysis of keystone microbial taxa

C. Within treatment group β-diversity comparisons

D. Effects of maternal CD4 count on microbial taxonomic α-diversity

E. Effects of cotrimoxazole treatment on reported illness and of reported illness on HEU-infant α-diversity

F. Comparisons to other investigations of HEU infants and of cotrimoxazole effects on microbiota

G. Overview of Supplementary Model Information Document

A. Microbial taxa and resistance gene α-diversity increases significantly over time in cotrimoxazole treated HEU (CTX-T) infants but not in HEU infants not treated with cotrimoxazole (CTX-N infants).

α-diversity measured by Shannon index, a diversity metric that accounts for microbial taxa evenness, showed similar trends to richness in the CTX-T infants for microbial taxonomic profiles (A-B p=0.033, A-C p=0.043; Wilcoxon signed-rank test), functional metabolic pathways (A-B p=0.015, A-C p=0.045; Wilcoxon signed-rank test), resistance genes (A-B p=0.0099, A-C NS; Wilcoxon signed-rank test), and *dfr*/*sul* resistance genes (A-B p=2e-5, A-C p=0.0043; Wilcoxon signed-rank test) (Supplemental Figure 2A-D). These significant α-diversity increases in CTX-T infants, but not in CTX-N infants may indicate bacterial response to cotrimoxazole selection pressure.

B. Cotrimoxazole treatment does not have a significant effect on keystone taxa.

Several bacteria taxa have been identified as keystone members of the human gut microbial community[1]. We investigated variation in several keystone taxa members over time to determine if cotrimoxazole treatment had significant effects on the relative abundance of these taxa (Phyla: Actinobacteria; Genus: *Bacteroides*, *Ruminococcus*, *Klebsiella*, *Proteus*) in the HEU infant gut microbiota. Linear mixed-effects modeling did not show significant differences in any of these taxa by cotrimoxazole treatment (Supplemental Model Information). There was also no significant difference in *Pseudomonadaceae* (Supplemental Model Information), a bacterial family identified by Bender *et al.* 2016 as a significant differentiator between HIV-exposed and HIV-unexposed infants[2].

C. CTX-T infants have sustained decreases in microbial taxa, functional metabolic pathway, and resistance gene β-diversity from 6 weeks to 4 months and to 6 months.

To understand the intersample β-diversity of the HEU infant gut microbiomes, we calculated pairwise Bray-Curtis dissimilarities[3] for our samples' microbial taxonomic profiles, functional metabolic pathways, and resistance genes. β-diversity is diversity between two samples and higher diversity indicates that sample compositions are more different from each other. Using these Bray-Curtis dissimilarities, we first investigated change in dissimilarity within treatment group over time (Supplemental Figure 7).

Under normal development conditions, infant microbial and functional β-diversity decreases over time relative to other infants and adults[4,5]. Low species counts in early-life contributes to high variation and consequently, high measured β-diversity. As individuals mature, they pick up additional species and these additional species serve to reduce variability in the gut microbiome. This reduced variability also reduces measured β-diversity. This maturation step often involves broadly shared selection pressures on the gut microbiota across different individuals (e.g. transition out of the womb to an open environment or transition to solid foods).

From timepoint A to timepoint B we observed significant decreases in β-diversity for both CTX-T and CTX-N infants for microbial taxonomic profiles (CTX-N $p=7e-4$; CTX-T $p<1e-5$; Wilcoxon signed-rank test), functional metabolic pathways (CTX-N $p=1e-5$; CTX-T $p<1e-5$; Wilcoxon signed-rank test), and resistance genes (CTX-N $p=1e-5$; CTX-T $p<1e-5$; Wilcoxon signed-rank test). These trends held true for all the treatment group timepoint A to timepoint C comparisons (all $p<1e-5$; Wilcoxon signed-rank test), but CTX-N infants were not significantly more similar in timepoint C compared to timepoint A in their microbial taxa or resistance gene β-diversity, though their functional pathway β-diversity continued to be significantly different ($p=0.0033$; Wilcoxon signed-rank test). These results show that taxonomic, functional pathway, and resistance gene β-diversity is significantly and lastingly reduced in CTX-T infants. This is potentially due to

bottlenecking following cotrimoxazole treatment selection pressures. Lower β-diversity in CTX-T infants compared to CTX-N infants for microbial taxonomic profiles, functional metabolic profiles, and resistance genes is consistent with cotrimoxazole selection pressure constricting HEU infant microbiomes. The effect sizes are larger for resistance genes and taxonomic profiles than for functional pathways, indicating functional pathways may have higher redundancy. It is notable that these results are mirrored in the functional pathways for CTX-N infants, but not in their taxonomic profiles or resistance genes.

Though the CTX-N infants are likely disturbed compared to HIV-unexposed infants, we still expected their β-diversity to decrease over time. The significant decreases for functional metabolic pathways could indicate that functional pathways have a redundancy across microbiomes that is not recapitulated in taxonomy and resistance genes. Bäckhed *et al.* 2015 looked at normal gut microbiome development in infants over the course of 1 year and found similar decreases in functional gene β-diversity[5]. Though they did not investigate resistance gene β-diversity, they presented decreases in microbial taxonomic β-diversity, but the decreases in β-diversity they show for microbial taxa are small and are given at the genus level.

D. Maternal CD4 count does not significantly alter HEU infant gut microbial taxa α-diversity.

Bender *et al.* 2016 showed lower α-diversity in HEU infants than HIV-unexposed infants when the HEU infants were born to mothers with low CD4 T cell counts (defined in this study as less than 350)[2]. They did not find α-diversity differences between HEU infants based on their maternal CD4 T cell counts. We used linear models to determine if microbial taxa α-diversity in our cohort was affected by maternal CD4 T cell count (Supplemental Figure 3). For both Shannon index and richness the strongest positive relationship with CD4 T cell count occurred at timepoint A (Shannon

index slope=0.0011, p=0.12; richness slope=0.021, p=0.36) and the weakest relationship occurred at timepoint C (Shannon index slope=-0.00022, p=0.77; richness slope=-0.00011, p=0.97). None of these relationships reached our threshold for statistical significance, and we also did not observe any significant differences by cotrimoxazole treatment.

E. Child GI symptoms or other illnesses were not associated with microbial taxonomic, resistance gene, or functional metabolic pathway α-diversity

We did not find significant differences between CTX-T and CTX-N infants with respect to incidence of all cause illness (Supplemental Figure 1A, p = 0.914; Fisher's Exact Test) or gastrointestinal specific (diarrhea or vomiting) illness (Supplemental Figure 1B; p = 0.447; Fisher's Exact Test) during the study period.

We next layered our clinical metadata onto our α-diversity calculations and found no effects from child all cause illness (Supplemental Figure 4) or gastrointestinal specific illness (Supplemental Figure 5) on microbial taxonomic, resistance gene, or functional metabolic pathway α-diversity measured by Shannon index and richness.

F. Comparisons to other investigations of HEU infants and of cotrimoxazole effects on microbiota

In Monaco *et al.* 2016, the authors looked at the effects of HIV-infection on the gut virome and the gut microbiome in a cohort of Ugandan adults. They 16S rRNA sequenced stools of 73 HIV-infected individuals (half on antiretrovirals and half naïve to antiretrovirals) and 37 HIV-negative controls. All but four of the HIV-infected individuals from this study were on long-term cotrimoxazole therapy. The authors compared the cotrimoxazole treated individuals to the untreated

HIV-uninfected controls to assess microbial taxonomic effects from the cotrimoxazole treatment and found only two differentially abundant OTUs.

Bourke *et al.* 2019 looked at effects of continuing or halting long term cotrimoxazole prophylaxis in HIV-infected Ugandan and Zimbabwean children between 4 and 11 years old. The authors whole-metagenome shotgun sequenced 72 samples at 84 weeks and 68 samples at 96 weeks post stopping treatment with approximately equal numbers of samples from the treatment and control groups in both timepoints. From this study, the authors did not find significant differences in α-diversity at the species level, but they did find 7 differentially abundant bacterial species between the two treatment groups. The authors then conducted additional analysis of the viridans group Streptococci, finding that several members of this group decreased with cotrimoxazole treatment.

Oldenburg *et al.* 2018 investigated effects of short courses of antimicrobials, including cotrimoxazole, on healthy preschool children's gut microbiomes collected via 16S rRNA sequencing of rectal swabs. 29 of the children analyzed from this cohort received cotrimoxazole for 5 days, and they were compared to the 29 control children. Samples were collected at baseline and 5 days following the end of treatment. α-diversity measured by inverse Simpson's index was not significantly different between cotrimoxazole treated children and placebo children.

In all three of these studies, the cohorts were significantly older than the cohort we present in this manuscript. Additionally, none of these cohorts were HIV-exposed, uninfected infants and only Bourke *et al.* 2019 conducted whole-metagenome shotgun sequencing on stool samples. Despite these cohort differences, none of these studies found significant differences in microbial taxonomic α-diversity following cotrimoxazole treatment. This result is consistent with our study where species richness and species Shannon index were both insignificant between CTX-T and CTX-N infants (Figure 6 and Supplemental Figure 6).

Bender *et al.* 2016 and Claassen-Weitz *et al.* 2018 both examined the effects of HIV exposure on the infant gut microbiome by comparing HEU infants to HIV-unexposed infants using 16S rRNA sequencing[2,6]. Bender and colleagues found HEU infants have perturbed microbiomes compared to HIV-unexposed infants. Specifically, HEU infants have lower stool α-diversity and higher prevalence of *Pseudomonadaceae* than HIV-unexposed infants. Bender *et al.* also found that maternal HIV status (CD4 count and viral load) affects HEU infant microbiota α-diversity. Claassen-Weitz and colleagues also looked at the effect of HIV exposure on infant stool α-diversity. Interestingly, HEU infants in their study had higher α-diversity than unexposed infants. This is the opposite of results from Bender *et al.*

Both studies found changes in α-diversity from HIV exposure. If this effect is strong enough it could mask potential effects from cotrimoxazole prophylaxis in our study. Since both studies were conducted with 16S rRNA rather than shotgun metagenomic sequencing, it was impossible to look at resistome characteristics in their cohorts. They also did not investigate the effects of cotrimoxazole prophylaxis on the infants. Since *Pseudomonadaceae* levels were different between exposed and unexposed infants in Bender *et al.*, we also looked at *Pseudomonadaceae* in our study. We only found *Pseudomonadaceae* in four CTX-T infants and in one CTX-N infant; this disparity was not statistically significant, and the low *Pseudomonadaceae* prevalence in our samples may reflect geographic differences.

Davis *et al.* 2017 and Lockman *et al.* 2017 looked at differences in health outcomes between cotrimoxazole treated and untreated HEU infants[7,8]. Davis *et al.* 2017 was an observational cohort study of 1984 infants that re-examined data from the BAN study[9] in malaria-endemic Malawi, and Lockman *et al.* 2017 was a randomized control trial of 2848 infants conducted in low malaria Botswana. Davis and colleagues found that cotrimoxazole prophylaxis significantly reduced both

respiratory and diarrheal morbidity. In contrast, Lockman and colleagues found no survival benefit from the cotrimoxazole prophylaxis. *E. coli* and *Klebsiella* spp. bacterial isolates from 220 infants of the cohort in Lockman *et al.* 2017[7] were analyzed in Powis *et al.* 2017[10]. They looked at antibiotic resistance in the bacterial isolates and found significantly higher cotrimoxazole resistance in bacteria from infant stools following cotrimoxazole treatment.

Similar to Botswana, South Africa does not have high malaria endemicity. Thus, infants in our cohort are unlikely to experience the highly significant morbidity reductions from cotrimoxazole prophylaxis reported in Davis *et al.* Instead they may receive no survival benefit as is reported in Lockman *et al.* 2017. The increases in bacterial isolate cotrimoxazole resistance found by Powis *et al.* is consistent with the increases in cotrimoxazole resistance genes we observe in our infant gut microbiomes.

G. Overview of Supplemental Model Information

This datafile includes all input commands and results for models included in this manuscript:

1. Cotrimoxazole effects on richness for
    a. Microbial taxa
    b. Functional metabolic pathways
    c. Resistance genes
    d. trimethoprim- and sulphonamide-resistance (*dfr*-*sul*) genes
2. Cotrimoxazole effects on Shannon diversity for
    a. Microbial taxa
    b. Functional metabolic pathways
    c. Resistance genes
    d. trimethoprim- and sulphonamide-resistance (*dfr*-*sul*) genes
3. Cotrimoxazole effects on keystone taxa
    a. Actinobacteria (phyla)
    b. Pseudomonadaceae (family)
    c. Bacteroides (genus)
    d. Ruminococcus (genus)
    e. Klebsiella (genus)
    f. Proteus (genus)
4. Cotrimoxazole effects on anthropometric measurements
    a. Weight
    b. Height
    c. Mid upper arm circumference
5. Clinical Metadata Models
    a. Child Unwell (all cause) vs. cotrimoxazole treatment contingency table and Fisher's exact test
    b. Gastrointestinal symptoms vs. cotrimoxazole treatment contingency table and Fisher's exact test

References

1. Banerjee S, Schlaeppi K, van der Heijden MGA. Keystone taxa as drivers of microbiome structure and functioning. Nat Rev Microbiol **2018**; 16(9): 567-76.
2. Bender JM, Li F, Martelly S, et al. Maternal HIV infection influences the microbiome of HIV-uninfected infants. Sci Transl Med **2016**; 8(349): 349ra100.
3. Bray JR, Curtis JT. An Ordination of the Upland Forest Communities of Southern Wisconsin. Ecological Monographs **1957**; 27(4): 325-49.
4. Yatsunenko T, Rey FE, Manary MJ, et al. Human gut microbiome viewed across age and geography. Nature **2012**; 486(7402): 222-7.
5. Backhed F, Roswall J, Peng Y, et al. Dynamics and Stabilization of the Human Gut Microbiome during the First Year of Life. Cell Host Microbe **2015**; 17(5): 690-703.
6. Claassen-Weitz S, Gardner-Lubbe S, Nicol P, et al. HIV-exposure, early life feeding practices and delivery mode impacts on faecal bacterial profiles in a South African birth cohort. Sci Rep **2018**; 8(1): 5078.
7. Lockman S, Hughes M, Powis K, et al. Effect of co-trimoxazole on mortality in HIV-exposed but uninfected children in Botswana (the Mpepu Study): a double-blind, randomised, placebo-controlled trial. Lancet Glob Health **2017**; 5(5): e491-e500.
8. Davis NL, Wiener J, Juliano JJ, et al. Co-trimoxazole Prophylaxis, Asymptomatic Malaria Parasitemia, and Infectious Morbidity in Human Immunodeficiency Virus-Exposed, Uninfected Infants in Malawi: The BAN Study. Clin Infect Dis **2017**; 65(4): 575-80.
9. van der Horst C, Chasela C, Ahmed Y, et al. Modifications of a large HIV prevention clinical trial to fit changing realities: a case study of the Breastfeeding, Antiretroviral, and Nutrition (BAN) protocol in Lilongwe, Malawi. Contemp Clin Trials **2009**; 30(1): 24-33.
10. Powis KM, Souda S, Lockman S, et al. Cotrimoxazole prophylaxis was associated with enteric commensal bacterial resistance among HIV-exposed infants in a randomized controlled trial, Botswana. J Int AIDS Soc **2017**; 20(3).
11. Oldenburg CE, Sie A, Coulibaly B, et al. Effect of Commonly Used Pediatric Antibiotics on Gut Microbial Diversity in Preschool Children in Burkina Faso: A Randomized Clinical Trial. Open Forum Infect Dis **2018**; 5(11): ofy289.
